# Application of power-law committee machine to combine five machine learning algorithms for enhanced oil recovery screening

**DOI:** 10.1038/s41598-024-59387-8

**Published:** 2024-04-22

**Authors:** Reza Yousefzadeh, Alireza Kazemi, Rashid S. Al-Maamari

**Affiliations:** Department of Petroleum and Chemical Engineering, College of Engineering, Sultan Qaboos University, Muscat, Oman

**Keywords:** Enhanced oil recovery, Committee machine, EOR screening, Machine learning, Planetary science, Solid Earth sciences

## Abstract

One of the main challenges in screening of enhanced oil recovery (EOR) techniques is the class imbalance problem, where the number of different EOR techniques is not equal. This problem hinders the generalization of the data-driven methods used to predict suitable EOR techniques for candidate reservoirs. The main purpose of this paper is to propose a novel approach to overcome the above challenge by taking advantage of the Power-Law Committee Machine (PLCM) technique optimized by Particle Swam Optimization (PSO) to combine the output of five cutting-edge machine learning methods with different types of learning algorithms. The PLCM method has not been used in previous studies for EOR screening. The machine learning models include the Artificial Neural Network (ANN), CatBoost, Random Forest (RF), K-Nearest Neighbors (KNN), and Support Vector Machine (SVM). The CatBoost is used for the first time in this work for screening of EOR methods. The role of the PSO is to find the optimal values for the coefficients and exponents of the power-law model. In this study, a bigger dataset than those in previous studies, including 2563 successful worldwide EOR experiences, was gathered. A bigger dataset improves the generalization of the data-driven methods and prevents overfitting. The hyperparameters of the individual machine-learning models were tuned using the fivefold cross-validation technique. The results showed that all the individual methods could predict the suitable EOR method for unseen cases with an average score of 0.868. Among the machine learning models, the KNN and SVM had the highest scores with a value of 0.894 and 0.892, respectively. Nonetheless, after combining the output of the models using the PLCM method, the score of the predictions improved to 0.963, which was a substantial increase. Finally, a feature importance analysis was conducted to find out the most influential parameters on the output. The novelty of this work is having shown the ability of the PLCM technique to construct an accurate model to overcome the class-imbalance issue in EOR screening by utilizing different types of data-driven models. According to feature importance analysis, oil gravity and formation porosity were recognized as the most influential parameters on EOR screening.

## Introduction

Over the past few decades, there has been a notable surge in global energy needs^1^. In reaction to this, oil and its derivatives have played a crucial role in meeting the world’s energy requirements. Projections indicate that this pattern is likely to persist in the forthcoming decades, with a rising demand for oil compared to alternative energy sources. Traditional methods of oil production from existing fields are inadequate to meet these escalating energy demands. Consequently, there is a growing call within the petroleum industry to enhance the ultimate recovery of mature and depleted oil fields. Unconventional resources, including shale and tight oil reservoirs, are becoming increasingly significant in addressing this challenge. Following the initial and secondary recovery phases, a significant amount of hydrocarbons is left behind in the reservoir.

Tertiary recovery, also known as Enhanced oil recovery (EOR), is employed to maximize the recovery factor and boost returns in oil and gas projects by focusing on mobilizing the remaining oil in the reservoir after primary and secondary recovery^2–4^. Unlike the primary and secondary recovery mechanisms, EOR entails fluid–fluid and fluid–rock physical and chemical interactions to facilitate the movement of trapped/remaining oil in the porous media. While EOR projects typically incur higher initial costs compared to traditional secondary projects, an ill-suited recovery project can result in lasting damage to the reservoirs and financial losses^5^. The process of finding the suitable EOR technique for a candidate reservoir involves various stages, including laboratory tests, reservoir characterization, simulation studies, pilot tests, and the final implementation of the method on the full-field scale. Each phase requires significant investments, and ignoring economically efficient screening poses risks to the success of the project. EOR screening serves as a pivotal step in risk reduction, offering the initial metric for evaluation with modest capital investment. Studies on EOR methods have shown that there are more than twenty types of different EOR techniques^6^ with and imbalanced frequency of the EOR techniques. Employing an inappropriate recovery technique could result in permanent damage to the reservoir and substantial financial losses and environmental hazards^7^. Therefore, a precise and dependable EOR screening process is crucial in the initial stages of reservoir development.

## Literature review

EOR screening approaches can be divided into two main categories: conventional EOR screening methods (CEORS) and advanced EOR screening methods (AEORS)^8,9^. CEORS relies on pre-specified screening of variables to assess the feasibility of implementing EOR techniques. Various parameters such as the properties of reservoir fluid and rock, including formation type, oil viscosity, permeability, oil saturation, layer thickness, oil gravity, salinity, reservoir type^10^, and depth, are crucial for the success of EOR methods^11,12^. These parameters can be used to assess the technical success of different EOR techniques for a specific field based on previously conducted projects. Nevertheless, a standardized methodology or universally accepted algorithm for the selection of a suitable EOR technique for a specific reservoir is still lacking. This is because of the complexity of reservoirs, encompassing factors such as anisotropy, heterogeneity, diverse rock-fluid interactions, geo-mechanical stress^13^, and numerous other unknown variables.

Although CEORS is studied by different authors, they lacked rigor and generality due to the challenges mentioned above. Consequently, AEORSs have been extensively used in the last decade to establish a rigorous relationship between the influential parameters and suitable EOR techniques for different types of reservoir models. Machine learning (ML) methods, such as multi-layer perceptron^14^, fuzzy inference^15^, Support Vector Machines (SVMs)^10^, etc., are some examples of AEORS techniques that are widely used for EOR screening. Khazali et al.^16^ employed a fuzzy decision tree (DT) trained on 548 successful EOR experiences related to ten different EOR techniques. Giro et al.^17^ correlated the physicochemical properties of the injected reservoir fluid with rock characteristics, including porosity, permeability, lithology, oil, water, and salt conditions and used a Naïve Bayes classifier to accomplish the EOR screening task. They collected 106 EOR samples with 15 different EOR categories and reached an accuracy of 0.90 on the test subset. However, their test subset did not include samples from all classes. Cheraghi et al.^6^ used shallow and deep Artificial Neural Network (ANN), Decision Tree (DT), Naïve Bayes (NB), and Random Forest (RF) to develop a model based on 1000 worldwide samples for EOR screening. They found that the RF performed the best with an accuracy of 0.91. Su et al.^18^ used the RF, ANN, NB, SVM, and DT for EOR screening of 13 EOR techniques as a function of porosity, permeability, depth, gravity, temperature, viscosity, net thickness, and initial oil saturation. They used 956 EOR experiences to train, validate, and test their models. Their results showed that the RF reached the highest accuracy of 0.91. Tabatabaei et al.^19^ used 281 EOR experiences with 20 different EOR categories to develop an ANN optimized by Particle Swam Optimization (PSO) and Sparrow Search Algorithm (SSA) to predict the most suitable EOR technique for the candidate reservoirs. Recently, Chavan et al.^10^ used 176 EOR projects to develop different data-driven models, including the ANN, SVM, KNN, Gaussian NB, and RF classifier to determine the most suitable EOR technique. They found that the RF outperformed the rest with an accuracy of 0.91.

Although many researchers have utilized different machine learning techniques for EOR screening, their works entail some limitations that necessitate the development of new methods to solve the problem. These challenges include the limited number of samples used to develop the models, using only one model to make the predictions, and not considering the previous production mechanism, which reduce the generalizability of the model. Table 1 summarizes the differences between the current study and previous similar studies with machine learning.

**Table 1:** Differences between our work and previous machine-learning-based studies.

Author(s)	Year	Method(s)	Differences with our work
Khazali et al.^16^	2019	Fuzzy DT	Relying on only one model, smaller dataset, not considering previous production mechanism, different ML models
Giro et al.^17^	2019	Naïve Bayes	Using only one model, small dataset, fewer EOR methods, not considering previous production mechanism, incomplete test subset, different ML models
Cheraghi et al.^6^	2021	ANN, DT, NB, RF	Compared four models, but did not integrate their outputs, smaller dataset, not considering previous production mechanism, different ML models
Su et al.^18^	2023	RF, ANN, NB, DT, SVM	No integration of the models, smaller dataset, fewer EOR methods, not considering previous production mechanism, different ML models
Tabatabaei et al.^19^	2023	ANN-PSO, ANN-SSA	Used only one model, limited number of samples, not considering previous production mechanism, different ML models
Chavan et al.^10^	2023	ANN, KNN, SVM, RF, NB	Compared five models, but did not integrate their outputs, smaller dataset, not considering previous production mechanism, different ML models

Consequently, this study proposes an innovative approach for AEORS which combines the output of five machine learning methods using the power-law committee machine (PLCM) approach, instead of relying solely on one model. Taking benefit from different types of machine learning algorithms, the proposed approach resolves the class-imbalance issue. Also, it utilizes an extra input variable and a bigger dataset than previous works, which inputs more information about the candidate reservoirs into the machine learning models and increases the generalization of the models. The machine learning methods include the ANN, CatBoost, SVM, KNN, and RF. The contribution of this paper is to examine the ability of the PLCM technique to optimally combine the predictions by five machine learning methods to overcome the class-imbalance problem. Imbalanced number of classes (EOR types) is a common challenge in screening of EOR techniques. In the end, this paper investigates the effect of input parameters on EOR screening as well and identifies the most and least influential parameters.

## Methods and materials

**Figure 1:**
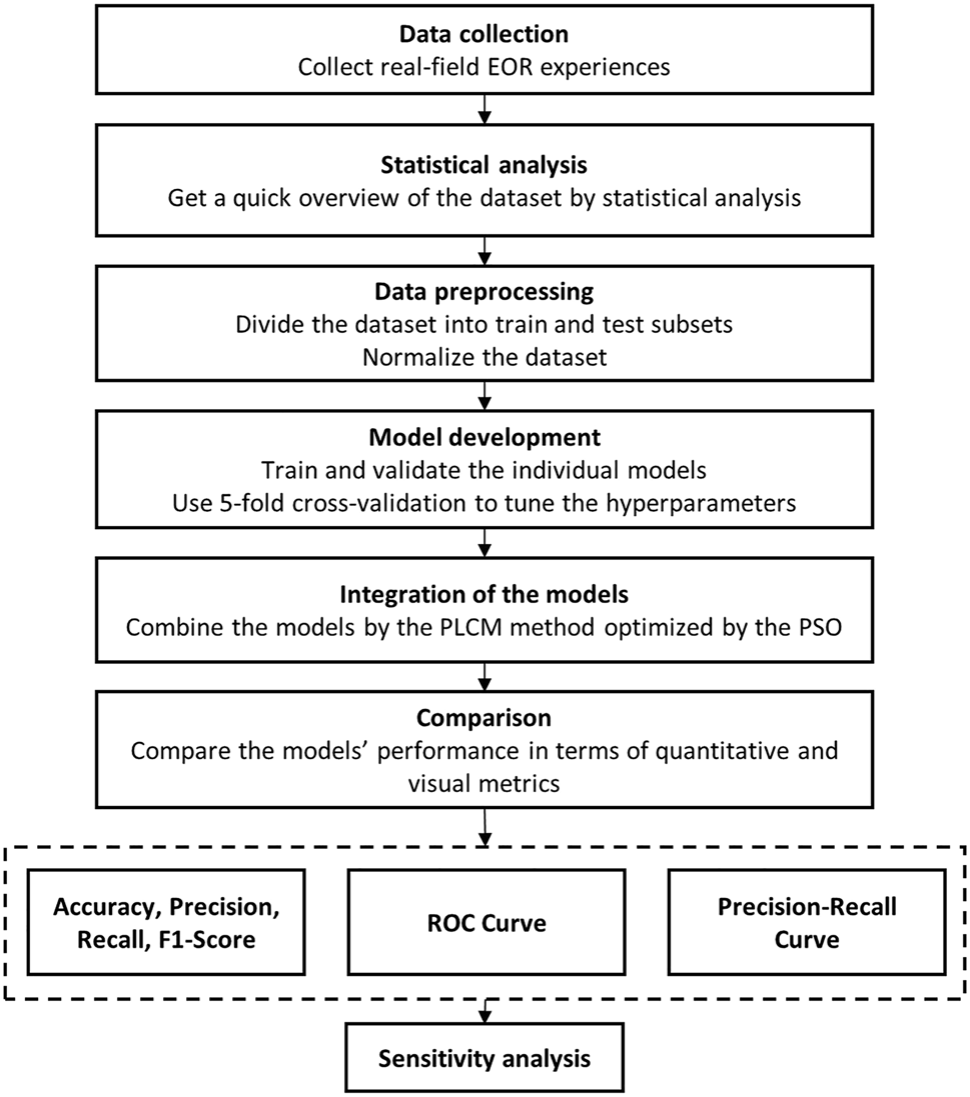
General flowchart of the study.

This study combines the predictions of five machine learning models by means of the PLCM method to increase the generalization of the model in the context of EOR screening. This study not only assesses the individual machine learning methods in predicting the most suitable EOR techniques, but also takes benefit from the PLCM method optimized by the PSO to increase the prediction accuracy, for the first time in the context of EOR screening. In this manner, the predictive tool is not limited to only one data-driven model, but also takes advantage of the strength points of different types of machine learning algorithms. Figure 1 shows the flowchart of this study. First, the required dataset to build and evaluate the utilized models is collected. Then, the data is preprocessed, which includes encoding the textual data into numeric values and normalizing the variables into [0,1]. Then, the individual machine learning models are trained. The hyperparameters of the models are tuned using a grid search with fivefold cross-validation. After training the individual models, their outputs are combined using the PLCM method optimized by the PSO algorithm. Then, the performance of the utilized methods is compared in terms of quantitative and visual evaluation metrics. The metrics, including the accuracy, precision, recall, F1-score, confusion matrix, precision-recall curve, and Receiver Operating Characteristic (ROC) curve to analyze their ability to handle the class imbalance issue. In the end, a feature importance analysis is conducted to find out the most influential input variables on the prediction of suitable EOR techniques. Another specialty of this study is that it uses a more comprehensive dataset than those in the previous studies, which increases the generalization of the developed model.

### Dataset

In this study, a dataset including 2563 EOR projects (available in Supplementary Information) from 23 different countries applied to sandstone, carbonate, limestone, dolomite, unconsolidated sandstone, and conglomerate reservoirs was collected from the literature^5,20–27^ to develop the screening methods. The utilized variables include the formation type, porosity (%), permeability (mD), depth (ft), viscosity (cP), oil gravity (API), temperature (°F), and the production mechanism before conducting EOR. The EOR techniques include injection of steam, hydrocarbon miscible, hydrocarbon immiscible, CO_2_ miscible, CO_2_ immiscible, carbonated water, low-salinity water, CO_2_ foam, nitrogen miscible, nitrogen immiscible, micellar polymer, surfactant/polymer, surfactant, cyclic steam drive, steam-assisted gas drive (SAGD), liquefied petroleum gas (LPG) miscible, in-situ combustion, polymer, alkaline/surfactant/polymer (ASP), hot water, microbial, air-foam, hydrocarbon miscible-WAG, and hydrocarbon immiscible-WAG. Table 2 reports the statistical analysis of the variables. Since formation is a categorical feature, it was converted to numerical values. Among fifteen different formation types, sandstone, carbonate, and dolomite are the most prevalent formation types with 45%, 10%, and 10% of the total data, respectively. To assess the accuracy of the developed models on unseen data, 85% of the data was used for training and the remaining 15% was used as blind test cases, and fivefold cross-validation is used for hyperparameter tuning. It is common to divide the dataset with a ratio of 70:15:15 as training, validation, and testing subsets. The validation subset is commonly used for tuning the hyperparameters of the models. Nonetheless, in the current study, 5-Fold cross validation was used to tune the hyperparameters, which does not require putting aside a portion of the data for validation. In this technique, the training subset is divided into K (5 in this study) non-overlapping folds. Then, the model is trained and validated K times with the fixed hyperparameters. One of the folds is used for validation and the others for training. Finally, the validation score is calculated as the average of scores over K repetitions. This is repeated for all configurations of the hyperparameters and the set of hyperparameters with the highest cross-validation score is selected. Thereby, as we did not need a separate validation subset, all samples, except for the testing subset, were used for training (85%).

**Table 2:** Statistical analysis of the utilized data.

	Formation	Porosity (%)	Permeability (mD)	Depth (ft)	Gravity (API)	Viscosity (cp)	Temperature (°F)
Mean	7	22.8	1366.8	4073.9	25.5	1.21E + 04	129.3
STD	4	10.1	1955.1	3019.9	12.1	2.10E + 05	46.8
Min	0	1	0	175	8	0.00E + 00	10
25%	3	12	35.25	1400	13	1.00E + 00	100
50%	9	25	614.5	3442	25	1.70E + 01	110
75%	9	31	2000	6000	37	1.90E + 03	158
Max	15	65	15,000	15,900	90	5.00E + 06	293

*STD* standard deviation

### Data preprocessing

One of the crucial steps before moving to model development is data preprocessing. One type of preprocessing is to encode textual values to numerical values, which is called label encoding. For example, the formation type, previous production mechanism, and EOR techniques are textual features, which were encoded as numbers. Another preprocessing step is scaling the data into similar intervals since the scale of the features differ significantly. For example, viscosity is in the order of 10^6^, while porosity is in the order of tens. In this study, the features were normalized into [0,1] interval using $(X - X_{\min } )/(X_{\max } - X_{\min } )$, where $X_{\min }$ and $X_{\max }$ are the minimum and maximum of the features in the training subset.

### Artificial neural network (ANN)

**Figure 2:**
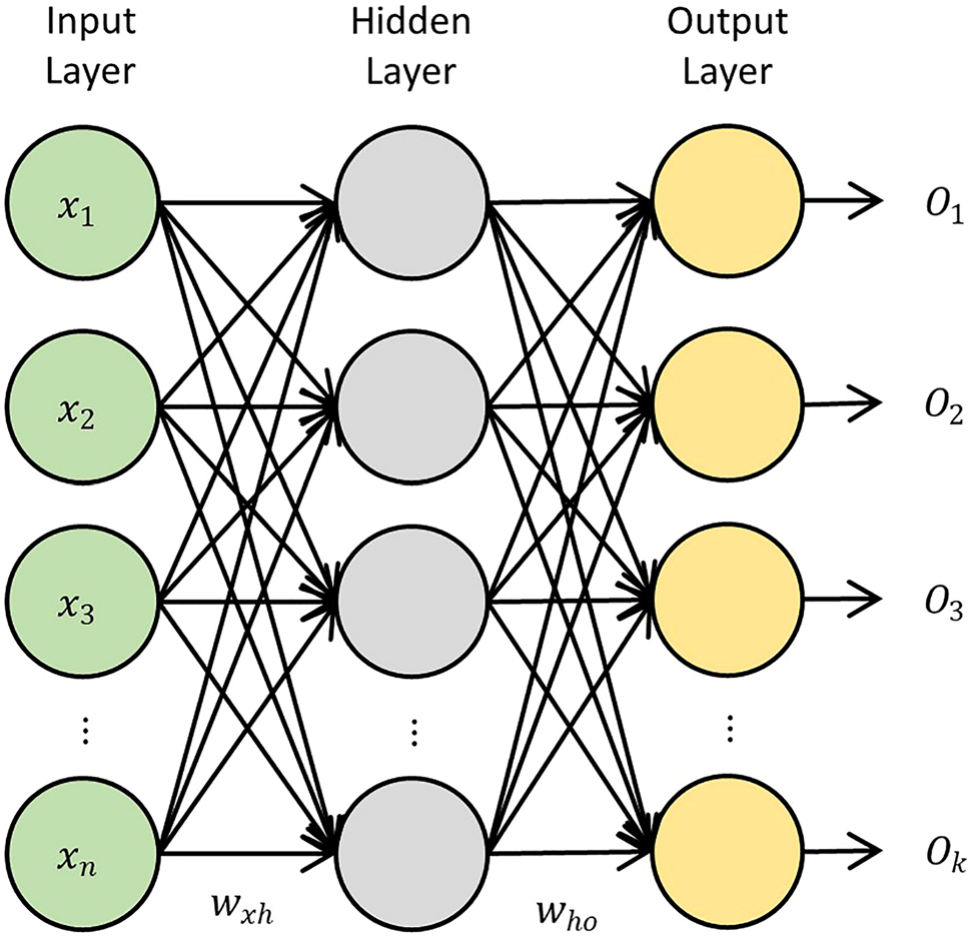
Schematic structure of an ANN.

ANN is a learning algorithm that is inspired by the human brain. ANN can figure out the relationship between the inputs and outputs without the need for complex mathematical or computational methods. Among the various types of ANN, the Multilayer Perceptron (MLP-ANN) stands out as the most commonly used^28–30^. The MLP includes three layers, namely input, hidden, and output layers^31,32^, as illustrated in Fig. 2. As shown, each layer consists of computational units known as neurons. The number of neurons in the input and output layers is the same as the dimension of the input and output variables, respectively. The number of hidden layers and their size should be determined by trial and error. Each neuron is connected to all neurons of the previous layers, which represents a unique linear combination of the data coming in from previous layer. The linear combination takes place using a set of weights. For example, $W_{xh}$ represents the set of weights mapping the inputs to the hidden layers, and $W_{ho}$ represents the set of weights mapping the hidden neurons to the output layer. Another critical aspect of an ANN model is the activation function, which receives the results of the linear combination, known as activations, and determines the activation of each neuron. Including hidden layers with non-linear activation functions in an ANN empowers it to capture non-linear dependencies. The weights are learned during the training phase of the model, which is the ultimate goal of the training process. Using these weights, the outputs, represented by $\hat{y}$, are calculated by the feed-forward process as below.

1 $$\hat{y} = f\left( {\mathop \sum \limits_{i = 1} W_{ij} x_{i} + b_{j} } \right),$$

where *f* is the activation function;  $b_{j}$ is the hidden layer bias; $x_{i}$ is the input for the *i*th variable; and, $W_{ij}$ is the connection weight between the *i*th input and *j*th neuron.

The learning process in an ANN is actually adjusting the weights and biases in the hidden layers using the backpropagation algorithm to minimize the loss function between the predicted and actual values^28,33^. In a multiclass classification problem, the outputs are converted to one-hot encoded vectors, where all elements of the vectors are zeros except for the element corresponding to that specific sample class. To handle multiclass classification, the categorical cross entropy is used as the loss function, which is defined as follows.

2 $$CCE\left( W \right) = \mathop \sum \limits_{i = 1}^{C - 1} y_{i} \log \left( {\hat{y}_{i} } \right),$$

where *y* denotes the vector of actual outputs and *C* is the number of classes. Each output in a multiclass problem is a vector of probabilities for each class. The probabilities are calculated using the Softmax activation function. To minimize the loss function, the gradient of the loss with respect to the weights and biases must be calculated and back propagated to all layers to update the weights. Given the gradient of the loss function, the weights can be updated as follows.

3 $$W^{t + 1} = W^{t} - \eta \nabla_{W} CCE,$$

where $W^{t + 1}$ and $W^{t}$ are the new and current weights, $\eta$ is the learning rate, and $\nabla_{W} CCE$ is the gradient of the loss function calculated by an optimization algorithm, such as Adam, Stochastic Gradient Descent (SGD), RMSprop, Adagrad, Momentum, Nestrov and Accelerated Gradient^34,35^.

ANNs offer a variety of hyperparameters that can be tuned to optimize the model’s performance. It includes options for controlling model structure, learning rates, and regularization. Furthermore, ANNs incorporate class weights into the loss function, addressing the problem of class-imbalance, which is useful for the problem understudy. It also supports multiclass classification. Accordingly, one of the utilized methods in this study is the ANN.

According to the explanations, the control parameters of the ANN are the number of hidden layers, number of neurons in the hidden layers, activation functions, the optimizer, and learning rate, which should be fine-tuned to achieve a satisfactory performance.

### CatBoost

**Figure 3:**
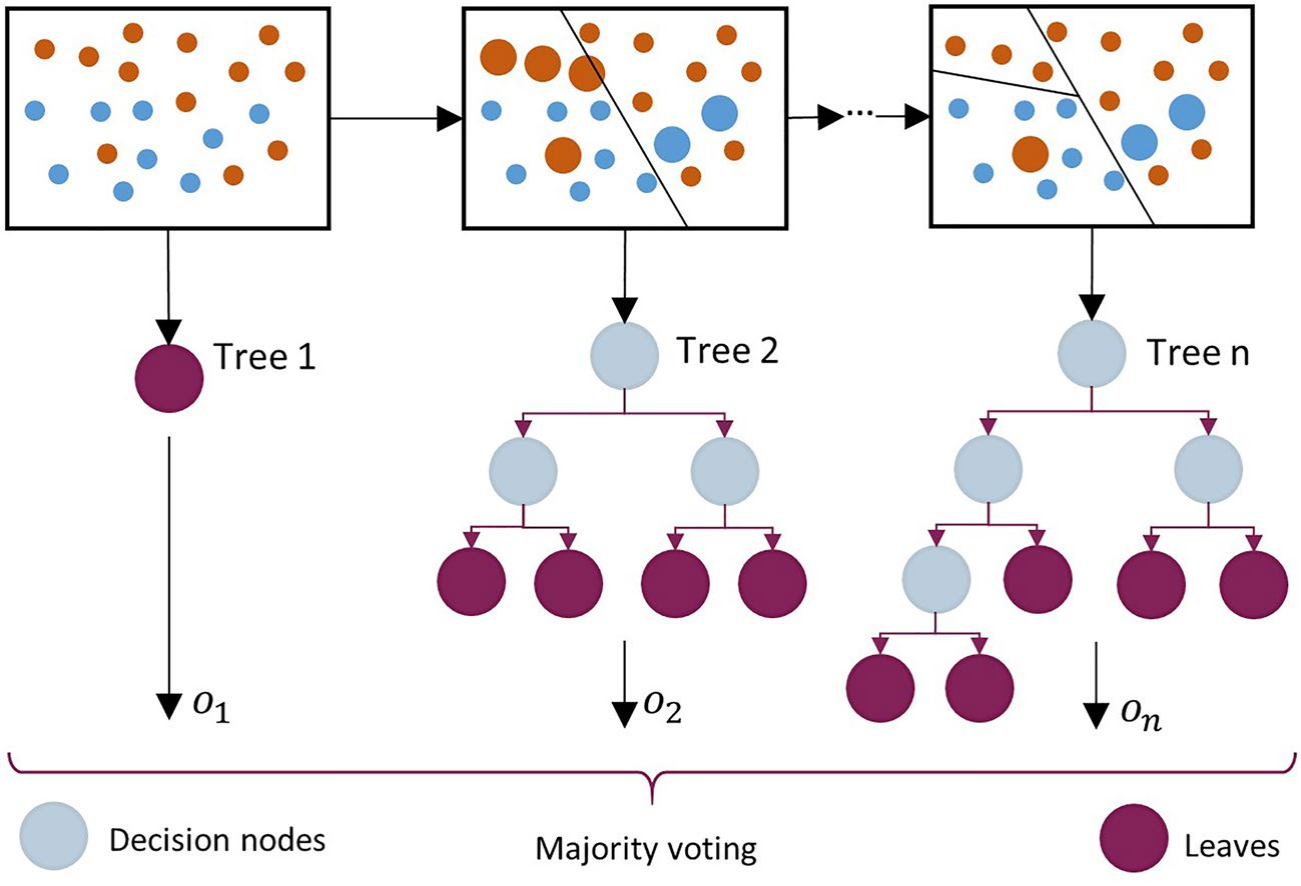
Schematic of the CatBoost tree construction.

CatBoost is a gradient-boosting tree construction method^36^, which makes use of both symmetric and non-symmetric construction methods. In CatBoost, a tree is learned at each iteration with the aim of reducing the error made by previous trees. Figure 3 shows the process of CatBoost tree building. In this figure, the orange and blue circles represent a dataset with two classes. The process starts with a simple initial model, assigning the average of the entire dataset to a single leaf node. Then, the misclassified samples (enlarged circles in Fig. 3) are identified and new trees are added based on the gradient boosting approach. Afterward, the predictions are updated to the combination of the predictions made by all trees. By adding new trees at each iteration, the number of misclassified samples decreases. Adding the trees continues until either the minimum number of samples required for splits or the maximum depth of the trees is reached. For categorical features, the CatBoost algorithm employs a symmetric splitting method for each feature. Then, based on the type of the feature, it chooses one of the split methods for each feature to create a new branch for each category^37^.

Considering a training dataset with $N$ samples, where $X$ is the matrix of inputs ($x_{1} ,\; \ldots ,\;x_{N}$) and $y$ is the vector of outputs ($y_{1} ,\; \ldots ,\;y_{N}$), the goal is to find a mapping function, $f(X)$, from the inputs to the outputs. Here, $f(X)$ is the boosted trees. Just like the ANN, the CatBoost needs a loss function ($L(f)$) to be minimized to perform the optimal tree building strategy.

Now, the learning process entails minimizing the $L(f)$.

4 $$f^{*} (X) = \arg \;\mathop {\min }\limits_{f} L\;(f) = \arg \;\mathop {\min }\limits_{f} \mathop \sum \limits_{i = 1}^{N} L\;(y_{i} ,\;\hat{y}_{i} ),$$

If the algorithm entails *M* gradient boosting steps, a new estimator *h*_*m*_ can be added to the model.

5 $$f_{m + 1} \;(x_{i} ) = f_{m} \;(x_{i} ) + h_{m} \;(x_{i} ),$$

where $f_{m + 1} \;(x_{i} )$ is the new model, and $h_{m} \;(x_{i} )$ is the newly added estimator. The new estimator is determined by employing the gradient boosting algorithm, where the steepest descent obtains $h_{m} = - \;\alpha_{m} g_{m}$ where $\alpha_{m}$ is the step length and $g_{m}$ is the gradient of the loss function.

Now, the addition of a new tree/estimator can be accomplished by

6 $$f_{m + 1} (x) = f_{m} (x) + \left( {\arg \mathop {\min }\limits_{{h_{m} \in H}} \left[ {\mathop \sum \limits_{i = 1}^{N} L\left( {y_{i} , \;f_{m} (x_{i} ) + h_{m} (x_{i} ) } \right)} \right]} \right)\;(x),$$

7 $$f_{m + 1} (x) = f_{m} (x) - \alpha_{m} g_{m} .$$

By taking benefit from the gradient boosting approach, the ensemble of decision trees built by the CatBoost algorithm often leads to a high prediction accuracy. The CatBoost also uses a strategy known as “ordered boosting” to improve the efficacy of its gradient-boosting process. In this type of boosting, a specific order is used to train the trees, which is determined by their feature importance. This prioritizes the most informative features, resulting in more accurate models^38^. The algorithm offers a wide range of regularization methods, such as depth regularization and feature combinations, which helps prevent overfitting. This is specifically useful when dealing with complex datasets.

The CatBoost offers a range of control parameters to optimize the structure of the model. These parameters include the number of estimators, maximum depth of the trees, maximum number of leaves, and regularization coefficients. These control parameters are optimized in this study to obtain the best performance from the model.

### K-nearest neighbors (KNN)

KNN is a non-parametric learning algorithm proposed by Fix and Hodges^39^. This algorithm does not have a training step and determines the output of a sample based on the output of the neighboring samples^10^. The number of neighbors is denoted by K. With K = 1, the label of the sample is as of the nearest sample. As the name of this algorithm implies, the K nearest neighbors are found based on the distance between the query sample and all samples in the dataset. Euclidean, Minkowski, Chebyshev, and Manhattan distances are some common distance measures. The Minkowski distance is a generalization of the Euclidean and the Manhattan distance with $p = 2$ and $p = 1$, respectively. *p* is the penalty term in L_p_ norm, which can be a positive integer. The distance between the samples greatly depends on the scale of the features. Therefore, feature scaling is of great importance^40^. After finding the K nearest samples to the new sample (query), its label is determined using Eq. (8).

8 $$\hat{f}(x_{q} ) \leftarrow {\text{arg }}\;\mathop {\max }\limits_{c \in C} \mathop \sum \limits_{i = 1}^{K} \delta (c, \;f(x_{i} )), \quad \delta (a,\;b) = 1 \quad {\text{if}}\;\; a = b.$$

where $x_{q}$ is the new sample, $f(x_{i} )$ is the label of the *i*th neighboring sample, *C* denotes the number of classes, and $\delta (a,\;b)$ is the Kronecker delta which is 1 if $a = b$ and 0 otherwise. An extension to KNN is the distance-weighted KNN, where the inverse of the distances between the samples are used as the weights. In this manner, the prediction for the query sample will be

9 $$\hat{f}(x_{q} ) \leftarrow {\text{arg }}\;\mathop {\max }\limits_{c \in C} \mathop \sum \limits_{i = 1}^{K} w_{i} \delta (c,\; f(x_{i} )),\quad \delta (a,\;b) = 1 \quad {\text{if}} \;\;a = b,$$

where $w_{i}$ is the inverse of the distance between the query sample and sample *i*, $w_{i} = 1/D(x_{q} ,\;x_{i} )$. Consequently, the closer neighbors will have a higher impact on the predicted label.

One distinctive feature of KNN that sets it apart from other machine learning methods is its ability to handle incomplete observations and noisy data^41^. This technique enables the identification of significant patterns within noisy data records. Another advantage of KNN is that it does not require any training and building and the model optimization can be done quite quickly. According to the above explanations, the controlling parameters of KNN are the number of neighbors (K), using/not using distance weighting, penalty terms, and the algorithm used to compute the nearest neighbors.

### Support vector machine (SVM)

**Figure 4:**
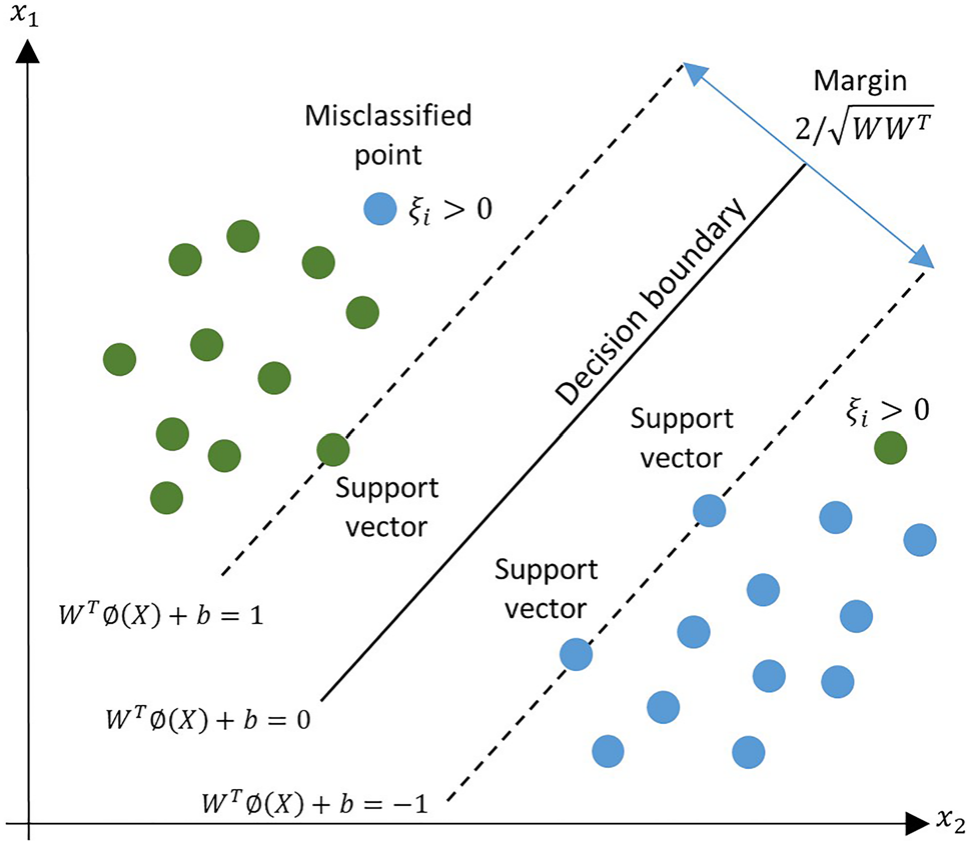
Schematic of a binary SVM.

SVM is a binary classification algorithm introduced by Cortes and Vapink^42^. SVM can be implemented to solve problems with linear or non-linear behavior^43,44^. However, non-linear data should be mapped into a higher-dimensional space to make it linearly separable. This technique is called the kernel trick. The classification is done by a decision boundary which has the maximum margin from both classes. Figure 4 shows the schematic of an SVM classifier for a binary classification task. The margins are constructed by finding the support vectors in each class and drawing the hyperplanes from the support vectors^45^. The hyperplanes are shown by dashed lines and the decision boundary is drawn between them. In this figure, the green circles represent the positive (+ 1) and the blue circles represent the negative (− 1) classes. The circles on the hyperplanes are the support vectors. The decision boundary with the maximum margin from the classes results in the highest generalization.

By considering the mapping function $\emptyset (X)$ and inputs $X$ and outputs $y$, the equation of the decision boundary can be written as follows^46^:

10 $$W^{T} \emptyset (X) + b = 0,$$

where *W* is the weight parameters and *b* is the bias term. The smallest perpendicular distance between the hyperplanes is known as the margin, which is double the distance between the support vectors and the decision boundary. Assuming that the data is separated by two hyperplanes with margin $\beta$, after rescaling *W* and *b* by $\beta /2$ in the equality, for each training example we have

11 $$y_{i} \left[ {W^{T} \emptyset (x_{i} ) + b} \right] \ge 1,\quad i = \left\{ {1,\;2, \ldots ,\;M} \right\}.$$

For every support vector ($X_{s} , \;y_{s}$) the above inequality is an equality. Thereby, the distance between each support vector and the decision boundary, *r*, is as follows

12 $$r = \frac{{y_{s} (W^{T} X_{s} + b)}}{\left\| W \right\|} = \frac{1}{\left\| W \right\|},$$

where $\left\| W \right\|$ is the L_2_ norm of the weights. Therefore, the margin between the two hyperplanes becomes $\frac{2}{\left\| W \right\|}$. The goal is to maximize $\frac{2}{\left\| W \right\|}$, which is equivalent to minimizing $\frac{1}{2}W^{T} W$. Consequently, the optimization problem of the SVM is:

13 $$\begin{gathered} \arg \;\mathop {\min }\limits_{W,b} \frac{1}{2}W^{T} W, \hfill \\ subject\; to \;y_{i} \left[ {W^{T} \emptyset (x_{i} ) + b} \right] \ge 1,\quad {\text{for}}\;\;i = 1,\; \ldots ,\;M. \hfill \\ \end{gathered}$$

Nonetheless, to increase the generalization of the model and avoid overfitting, slack variables ($\xi$)^46,47^ are used (see Fig. 3), which allow the model to have some miss-classified samples during training. This approach is known as the soft margin approach. Now, the optimization problem becomes

14 $$\begin{gathered} \arg \;\mathop {\min }\limits_{W,b} \left( {\frac{1}{2}W^{T} W + c\mathop \sum \limits_{i} \xi_{i} } \right), \hfill \\ subject\; to\; y_{i} \left[ {W^{T} \emptyset (x_{i} ) + b} \right] \ge 1 - \xi_{i} ,\quad {\text{for}}\;\;i = 1,\; \ldots ,\;M. \hfill \\ \end{gathered}$$

where *c* is a regularization factor that controls the weight of the slack variables in the loss function. Equation (14) is a dual optimization problem, which is solved using the Lagrange approach. The Lagrange approach converts a dual-optimization problem to a standard one by incorporating the equality and inequality constraints to the loss function. Thereby, Eq. (14) becomes

15 $$\begin{gathered} L(W,\;b,\;\alpha ) = \frac{1}{2}W^{T} W - \mathop \sum \limits_{i = 1}^{M} \alpha_{i} \left[ {y_{i} \left( {W^{T} \emptyset (X_{i} ) + b} \right) - 1} \right], \hfill \\ subject\; to \;\;0 \le \alpha_{i} \le c,\quad i = 1,\; \ldots ,\;M. \hfill \\ \end{gathered}$$

where $\alpha_{i}$s are Lagrange multipliers. To minimize the above loss function, its derivatives with respect to *W* and *b* are set equal to zero. By doing this, we obtain $W = \sum\nolimits_{i = 1}^{M} {\alpha_{i} y_{i} \emptyset (X_{i} )}$ and $\sum\nolimits_{i = 1}^{M} {\alpha_{i} y_{i} = 0}$. Plugging these back into the Lagrange gives the dual formulation.

16 $$\begin{gathered} \arg \;\mathop {\max }\limits_{\alpha } - \frac{1}{2}\mathop \sum \limits_{i,j = 1}^{M} \alpha_{i} \alpha_{j} y_{i} y_{j} \emptyset (X_{i} )\emptyset (X_{j} ) + \mathop \sum \limits_{i = 1}^{M} \alpha_{i} , \hfill \\ subject\;\; to\; \mathop \sum \limits_{i = 1}^{M} \alpha_{i} y_{i} = 0, \;\;0 \le \alpha_{i} \le c, \;\;i = 1,\; \ldots ,\;M. \hfill \\ \end{gathered}$$

Equation (16) is solved using a Quadratic Programming solver to obtain the Lagrange multipliers $\alpha_{i}$. $\alpha_{i}$ is non-zero only for the support vectors. Parameter *b* does not appear in the dual formulation, so it is determined separately from the initial constraints. Calculating $\emptyset (X_{i} )\emptyset (X_{j} )$ is computationally expensive since it requires two mapping operations and one multiplication, especially if the data is high-dimensional. To tackle this problem, the Kernel trick is introduced, where $\emptyset (X_{i} )\emptyset (X_{j} )$ is represented as a kernel function $K(X_{i} ,\;X_{j} )$ based on the Mercer’s Theorem^48^. Finally, after determining the Lagrange multipliers, the prediction for a new sample z is calculated as follows

17 $$y = sign\left( {\mathop \sum \limits_{i = 1}^{n} \alpha_{i} y_{i} K(X_{i,} z) + b} \right).$$

The kernel function should be determined by trial and error. Some of the commonly used kernels are the linear, polynomial, and radial basis function (RBF) kernels.

SVM is one of the most successful machine learning algorithms in hand-written digit recognition^49,50^. SVMs can handle high-dimensional data, making them suitable for tasks with a large number of features. Because of taking benefit from the maximum margin theory and slack variables, SVMs are resistant to overfitting. One special feature of the SVMs, making them different than other artificial intelligence tools, is the kernel trick that enables SVMs to solve different kinds of non-linear classification problems. The convex nature of the loss function of the SVM leads to a convex optimization problem, which ensures converging to a global optimum. Finally, memory efficiency due to using only support vectors to construct the model and ability to handle class-imbalance by incorporating the class weights to the loss function are two other advantages of the SVMs making them suitable for the EOR screening problem in this study.

According to above explanations, some of the most important control parameters of the SVM are the kernel function, regularization factor (*c*), the degree of polynomial kernels, the intercept of polynomial kernels (coef0), and class weights. Class weights are used to tackle the class-imbalance issue by giving larger weights to rare classes in calculating the loss function.

Since SVM is a binary classifier, to perform multi-class classification, one-to-rest or one-to-one approaches are used. In this study, the one-to-rest approach is used, where $C$ SVM models are trained. Each SVM model predicts membership of the samples in one of the *C* classes.

### Random forest

In the context of machine learning, Random Forest (RF) is an ensemble learning technique that builds a multitude of decision trees during training and combines their outputs to make more accurate and robust predictions^51^. RF is a supervised learning method, suitable for classification and regression tasks. Each tree in the forest is constructed independently, using a random subset of the features and samples with replacement from the training data^52^. This randomness adds diversity to the decision-making process, preventing the model from too much focusing on idiosyncrasies in the data. An RF takes a random approach to selecting a subset of input variables/features (controlled by the maximum number of features), and performs the optimal split to divide a node based on a split criterion. Avoiding tree pruning ensures maximal tree growth. As a result, a multitude of trees are constructed, and the model employs a voting mechanism to determine the most prevalent class in a classification task.

**Figure 5:**
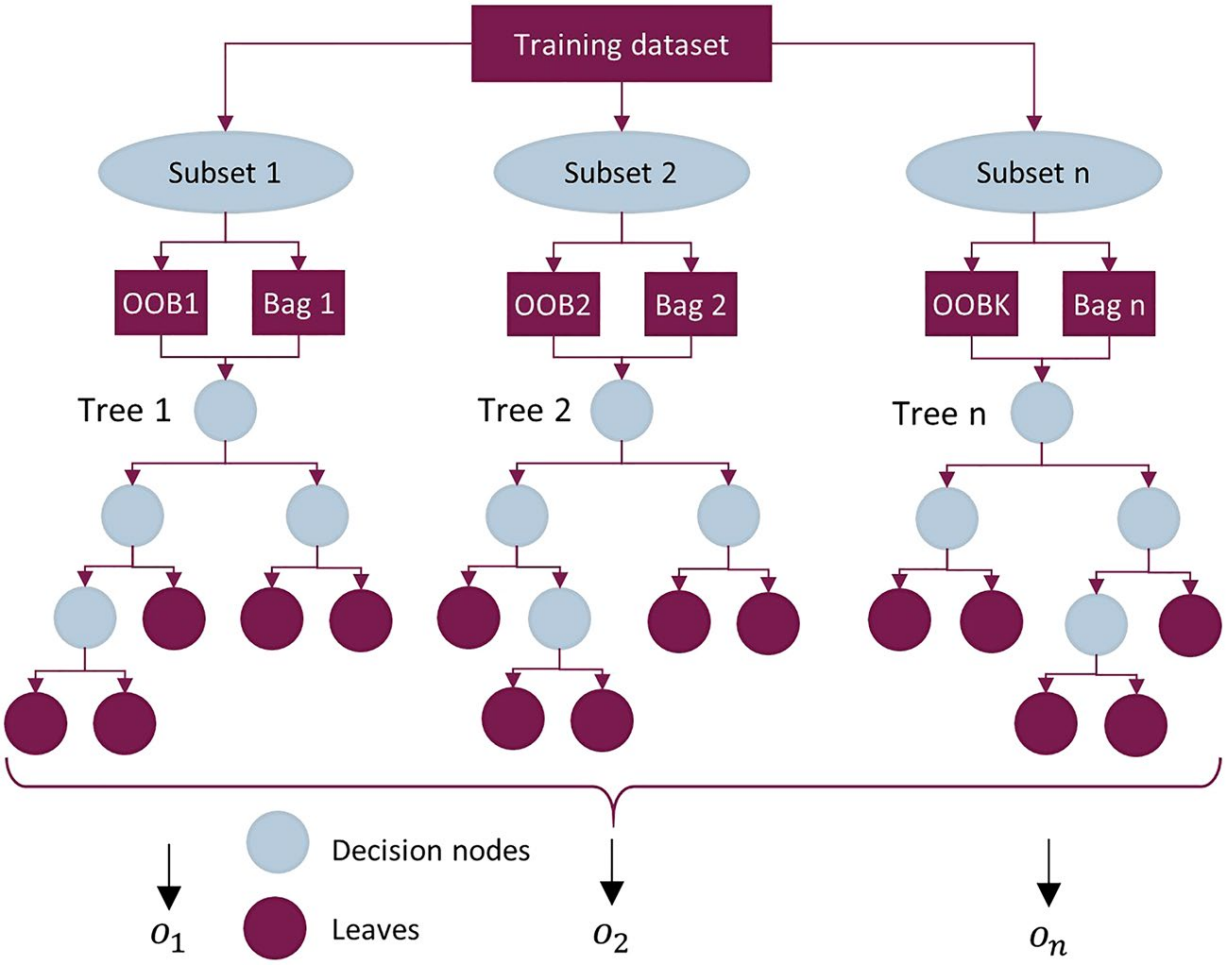
Schematic of the random forest tree construction.

Each tree makes its own prediction, and the final decision is determined by the majority voting paradigm. This approach not only enhances the prediction accuracy of the model but also makes it stronger against overfitting. Figure 5 shows the schematic of a random forest where *n* trees are used to make a prediction. Each subset is randomly selected from the dataset and divided into two parts, including the bag and out-of-bag (OOB) parts. The data in each bag is used to build a tree and the data in OOB is used to test that tree. The OOB subset serves as an ongoing and unbiased estimation of the general prediction error, predating the verification of prediction accuracy through the independent testing subset for the aggregated results. When $X$ is inputted to the ensemble, each tree provides a separate output ($o_{1} ,\; \ldots , \;o_{n}$). In the end, the ultimate class of the inputs is determined by the same approach given in Eq. (8).

The RF produces competing results to boosting and bagging, without any alteration to the training set. It minimizes the bias by incorporating a random sample predictor before each node segmentation. The RF model can handle high-dimensional data, without need for feature selection. Its implementation in Python is relatively straightforward, boosting training speeds and easy parallelization. Given these advantages, it is becoming increasingly popular among data scientists^52,53^.

According to the above explanations, the control parameters of a random forest are the split criterion, maximum depth of trees, the number of estimators, and the maximum number of features. These control parameters are fine-tuned to achieve the best performance. There is also another control parameter, which is the minimum number of samples required to split a node, but it is not investigated in this study.

### Power-law committee machine (PLCM)

A committee machine is a technique to merge the output of a multitude of predictive models to come up with a single prediction^33^. The benefit of this technique is to take advantage of the results of different alternatives for modeling a particular problem, instead of using only one model. The individual models are selected in such a way that at least one model from each type of machine learning models is included. Thereby, we can take benefit from the strength points of different types of learning algorithms. By using the PLCM technique, the chance of overfitting can be lowered^33^. There are two main approaches to combine the output of individual models, namely the static and dynamic approaches. In the static method, a linear combination of the individual outputs is used to get the ultimate output, while the dynamic approach uses a non-linear combination of the outputs. In this study, the dynamic approach with a power-law model is used to accomplish the integration task. Equation (18) shows the power-law model.

18 $$y = \mathop \sum \limits_{i = 1}^{5} \alpha_{i} y_{i}^{{\beta_{i} }} ,$$

where $y$ is the ultimate output, $\alpha_{i}$ and $\beta_{i}$ are the coefficients that must be optimized to achieve the goal of the power-law committee machine, and $y_{i}$ is the output of the $i$-th individual predictive model. In this study, the coefficients of the power-law model ($\alpha_{i}$ and $\beta_{i}$) are optimized by the PSO algorithm to achieve a satisfactory integration of the outputs. The PSO is described in the following subsection.

### Particle swarm optimization (PSO)

Kennedy and Eberhart^54^ introduced the PSO as a population-based optimization algorithm. This algorithm starts solving the problem with random solutions^65^. Each solution in this algorithm is known as a particle, where a swarm is composed of a multitude of particles. The particles change their position in the solution space by a specified velocity which is updated at each iteration. The particle’s position determines the solution found by the particle. When the position of the particle changes, a new solution is obtained. The following equations give the updating formulae for the velocity and position of a particle

19 $$v_{i} (t + 1) = \omega v_{i} (t) + c_{1} r_{1} (x_{best,i} (t) - x_{i} (t)) + c_{2} r_{2} (x_{best,g} (t) - x_{i} (t)),$$

20 $$x_{i} (t + 1) = x_{i} (t) + v_{i} (t + 1),$$

where $x_{i}$ and $v_{i}$ are the position and velocity of particle $i$, respectively, $t$ is the iteration number, $\omega$ is the inertia coefficient, $c_{1}$ and $c_{2}$ are the self-learning and social-learning coefficient, respectively, $r_{1}$ and $r_{2}$ are two random numbers, $x_{best,i}$ is the best solution found by the particle, and $x_{best,g}$ is the global best solution. The values of the $x_{best,i}$ and $x_{best,g}$ are obtained by evaluating the objective function. In this study, the objective function is the negative of prediction accuracy by the PLCM method. The velocity and position of the particles are updated until the algorithm reaches the stopping criterion. The parameters used in Eq. (19) are determined based on the work by Poli et al.^56^, where $\omega ,$ $c_{1} ,$ and $c_{2}$ are set at 0.7298, 1.49618, and 1.49618, respectively.

The PSO is one of the most commonly used optimization algorithms in petroleum engineering^57–60^. Among different metaheuristic optimization algorithms, the PSO has shown a better performance compared to the most of other optimization algorithms, such as the genetic algorithm and simulated annealing. The PSO has shown the ability to reach better optimal solutions and faster convergence to similar results than its rivals in many applications^61^. Thereby, this algorithm is used in this study to optimize the coefficients of the PLCM method.

After describing the tools used in this study, it is necessary to define the evaluation metrics, which are required to evaluate the performance of the proposed method. These metrics include the quantitative and visual indicators that are described in the following subsection.

### Evaluation metrics

In this study, quantitative and visual evaluation metrics are used to assess the performance of the proposed method. These metrics include the accuracy, precision, recall, F1-score, confusion matrix, Receiver Operating Characteristic (ROC) curve, and precision-recall curve.

#### Accuracy

Accuracy is the total number of correct predictions divided by the total number of data points. In binary classification, accuracy is defined as the number of true positives (TP) divided by the number of samples $accuracy = \frac{TP}{N}$, where N is the total number of data points/samples.

#### Precision

Precision is the portion of positive predictions that are actual positives. Precision focuses on the accuracy of positive predictions. For a binary classification precision is defined as $Precision = \frac{TP}{{TP + FP}}$, where FP is the number of false positives, which means that the prediction by the model is positive, whereas the actual label of the sample is negative.

#### Recall

Recall gives the portion of the positive samples that are identified as positives. Recall focuses on how well the model captures positive instances. In other words, it is the ratio of true positives to all positive samples in the dataset defined as ${\text{Re}} call = \frac{TP}{{TP + FN}}$, where FN is the number of false negative predictions defined as the samples which are incorrectly classified as negative.

#### F1-Score

The inverse of the harmonic average of the recall and precision multiplied by 2 is known as F1-Score. F1-Score is defined in Eq. (21).

21 $$F1{ - }Score = 2\frac{PR}{{P + R}},$$

where P and R are the precision and recall, respectively. A good classifier should have high values of precision and recall, which indicates a high F1-Score.

In multi-class classification, as the problem in this study, each metric is calculated for individual classes and averaged across all classes to obtain a single value. In this manner, each time, one of the classes is considered positive, and other classes are assumed as negative.

#### Confusion matrix

In a multiclass problem, the confusion matrix is a $C \times C$ matrix, where the rows represent the actual class and the columns represent the predicted class of the samples. The values on the main diagonal of the matrix show the number of correct predictions (true positives), and off-diagonal values show the number of incorrect predictions (false positives). The sum of the values on the main diagonal of the matrix divided the total number of samples gives the accuracy, as described above. Also, the diagonal value for each class if divided by the sum of all values in each column gives the class-specific precision, and if divided by the sum of all values in each row gives the class-specific recall.

#### Receiver operating characteristic (ROC) curve

ROC curve is a graphical representation that demonstrates the performance of a binary classification model across various thresholds. The threshold refers to the decision boundary that the predictive model utilizes to classify samples into one of the classes. It plots the true positive rate (sensitivity or recall) versus the false positive rate (1 − specificity) at various threshold values.

The key components of a ROC curve are:

- True Positive Rate (TPR): This is the portion of actual positive samples that are correctly classified by the classifier. It is calculated as TP/(TP + FN).
- False Positive Rate (FPR): This is the proportion of actual negative samples incorrectly classified as positive by the classifier. It is calculated as FP/(FP + TN), where TN is the number of true negatives.

The ROC curve is drawn by plotting the TPR against FPR, typically at different threshold values. A diagonal line, known as the random line, represents a random classifier. It is expected that the ROC curve of a good classifier bends towards the top-left corner of the plot, indicating a high recall and high specificity (low false positives) simultaneously.

To summarize the overall performance of a binary classifier, the area under the ROC curve (AUC) is used. A higher AUC value indicates better classification ability of the model across different threshold settings. For a multiclass problem, the ROC curve is created for each class and the AUC of each curve is calculated. Another way is to calculate the TPR and FPR for all classes and use their average value to create a single ROC curve with a single AUC value. In this study, the former approach is used. This approach provides the ability to analyze the performance of a model on each class.

#### Precision-recall curve

The precision-recall curve is another graphical representation of a binary classifier’s performance. Similar to the ROC curve, the precision-recall curve is generated by changing the threshold of the classifier and plotting the precision against recall at different thresholds. The curve shows a trade-off between precision and recall.

The benefit of a precision-recall curve is bolstered when dealing with imbalanced datasets, where one class (usually the negative class) significantly outnumbers the other (positive) class. In such cases, relying on the accuracy alone is not enough and precision and recall provide a more informative analysis of the classifier’s performance. A good classifier is expected to have a precision-recall curve that bends towards the top-right corner of the plot.

Similar to the ROC curve, the area under the precision-recall curve is used to quantify the overall performance of a classifier. A higher AUC value indicates higher precision and recall values simultaneously across different thresholds. An AUC of 0.5, which corresponds to the diagonal line in the plot, represents a random classifier.

## Results and discussion

In this section, the results of the individual predictive models along with the results of the PLCM method are presented and discussed. To tune the hyperparameters of the individual models, fivefold cross-validation is used. The optimal hyperparameters and their investigation ranges are reported for each method.

Table 3 shows the results of hyperparameter tuning along with the investigation ranges and the average cross-validation score for the best set of hyperparameters for each method. As the results show, all the methods have reached satisfactory cross-validation scores (accuracy), ranging from 0.870 to 0.883. In addition to cross-validation results, the performance of the methods on training and testing subsets is reported in Table 4 The methods are evaluated based on accuracy, recall, precision, and F1-Score.

**Table 3:** Optimal hyperparameters and their investigated ranges with cross-validation scores for each method.

Method	Hyperparameter	Range	Optimal value	Cross-validation score
ANN	No. of layers	1–4	3	0.883
	No. of neurons	50, 100, 200	100	
	Activation function	relu, tanh, elu	tanh	
	Learning rate	0.001, 0.01	0.01	
	Optimizer	SGD, Adam	Adam	
CatBoost	Max. depth	2, 3, 4	3	0.873
	Max. leaves	10, 31, 50	31	
	No. of estimators	10–100	20	
	Leaf regularization	0, 5, 10	0	
KNN	Algorithm	“auto”, “ball_tree”, “kd_tree”, “brute”	auto	0.886
	No. of neighbors	1, 3, 5, 7, 9	5	
	Penalty	1, 2	1	
	Weights	Uniform, distance	Distance	
SVM	C	1, 10, 100, 1000	100	0.870
	Class weight	Balanced, none	None	
	coef0	0, 1, 10	0	
	Degree	None, 2, 3, 4, 5	None	
	Kernel	“rbf”, “poly”	rbf	
RF	Criterion	‘gini’, ‘entropy’, ‘log_loss’	Entropy	0.867
	Max. depth	1, 2, 3	3	
	Max. features	“sqrt”, “log2”, 1, 2	1	
	No. of estimators	10–100	40

**Table 4:** Performance of the individual methods on the training and testing subsets.

Method	Subset	Accuracy	Precision	Recall	F1-Score
ANN	Train	0.926	0.898	0.926	0.911
	Test	0.893	0.850	0.893	0.868
CatBoost	Train	0.907	0.873	0.907	0.889
	Test	0.872	0.806	0.872	0.836
KNN	Train	0.997	0.998	0.997	0.997
	Test	0.906	0.891	0.906	0.894
SVM	Train	0.983	0.981	0.983	0.981
	Test	0.906	0.882	0.906	0.892
RF	Train	0.909	0.886	0.908	0.896
	Test	0.880	0.828	0.880	0.850

**Figure 6:**
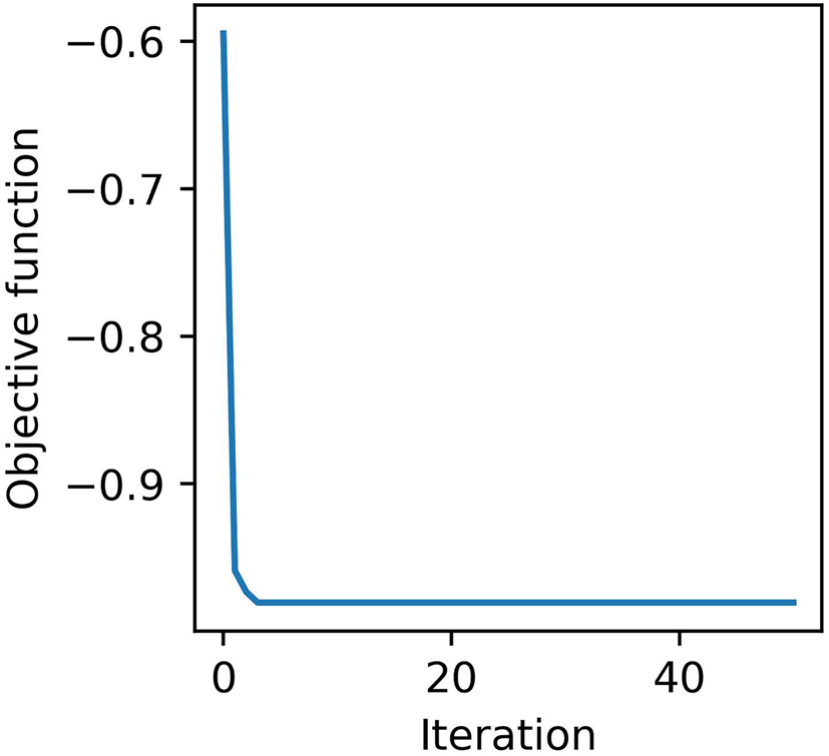
Classification error during optimization of the PLCM model.

As presented in Table 4, all the methods have shown reliable performance in predicting the suitable EOR techniques for the training and testing samples. Consequently, they are eligible to construct the PLCM model. The PSO was used with 50 particles and 50 iterations to optimize the coefficients of the PLCM model. Figure 6 shows the classification error during the optimization process of the PLCM model. As can be seen, the error has significantly reduced, indicating that the PSO algorithm has been successful at optimizing the coefficients of the PLCM model. Tables 5 and 6 show the optimal coefficients of the PLCM model and its performance on the training and testing subsets, respectively.

**Table 5:** Coefficients of the PLCM method.

$\alpha_{1}$	$\beta_{1}$	$\alpha_{2}$	$\beta_{2}$	$\alpha_{3}$	$\beta_{3}$	$\alpha_{4}$	$\beta_{4}$	$\alpha_{5}$	$\beta_{5}$
0.967	0.098	0.0012	0.893	0.532	1.183	0.036	0.979	0.030	0.539

**Table 6:** Performance of the PLCM model on the training and testing subsets.

Metric	Subset	
	Train	Test
Accuracy	0.980	0.963
Precision	0.980	0.964
Recall	0.980	0.963
F1-Score	0.980	0.963

**Figure 7:**
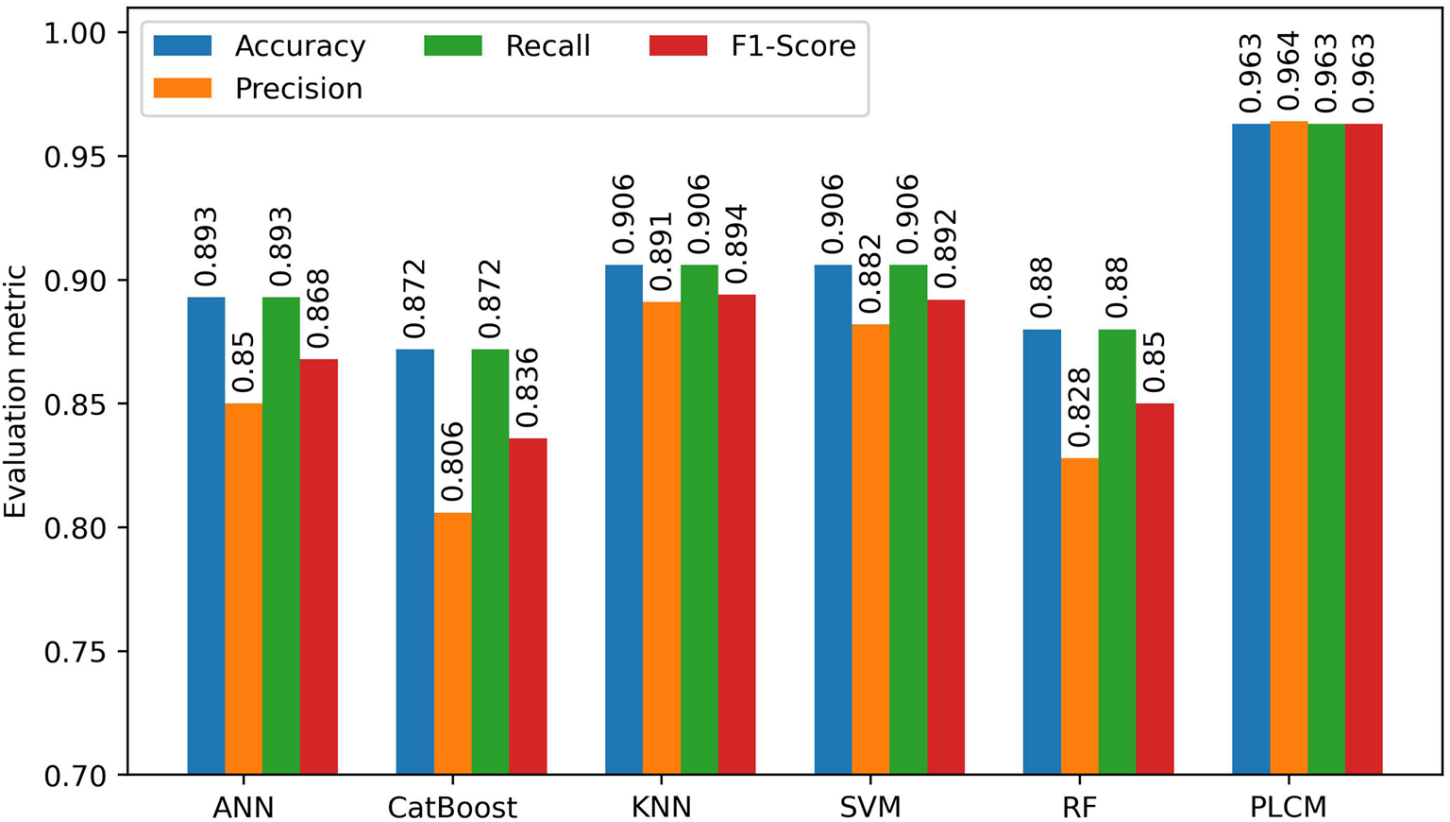
Evaluation metrics for all utilized methods for EOR screening.

According to Table 5, the PLCM method has shown outstanding performance at attributing the correct EOR method to the samples in the training and testing subsets. In addition, merging the results of the individual models using the power-law committee machine has significantly improved the prediction metrics. To have a clearer comparison between the individual methods and the PLCM model, their average evaluation metrics on all classes of the test subset are shown in Fig. 7. As shown in Fig. 7, there is an evident gap between the evaluation metrics of the PLCM model with the evaluation metrics of the individual models. Also, all metrics are high and close to each other, indicating that the PLCM technique can correctly identify positive and negative classes. Although the KNN model has better metric than the PLCM model on the training subset, its metrics on the testing subset are substantially lower than those of the PLCM model, which indicates the potential overfitting of the KNN model. The worst performance goes to the CatBoost algorithm since its F1-Score is the lowest among all. Its low F1-Score is related to its low precision (0.806) indicating that the CatBoost algorithm had many false positives. The second worst model was the RF with an F1-Score of 0.850, again related to its low precision. As a result, tree-based algorithms had a high rate of false positives, meaning that they had many incorrect positive predictions for specific samples. After the tree-based methods, ANN had a moderate performance with an F1-Score of 0.868. This method showed better precision and recall than the tree-based methods, which is the reason for its higher F1-Score. The best individual models are the KNN and SVM models with F1-Scores of 0.894 and 0.892, respectively. In the meanwhile, KNN showed a higher precision than the SVM. Thereby, KNN is slightly better at correctly identifying the true positives. This means that when the KNN says that the class of a sample is A, with a high probability the true class is A. However, none of the individual methods could achieve an F1-Score higher than 0.90. The low precision of the individual methods indicates that they lack rigor to correctly predict actual positive samples as positives. Nonetheless, merging the outputs of the individual models has resolved this issue and improved the predictions, achieving an F1-Score of 0.963 and precision of 0.964. This is because of taking benefit from the strength of each individual model optimally to overcome the class imbalance issue.

**Figure 8:**
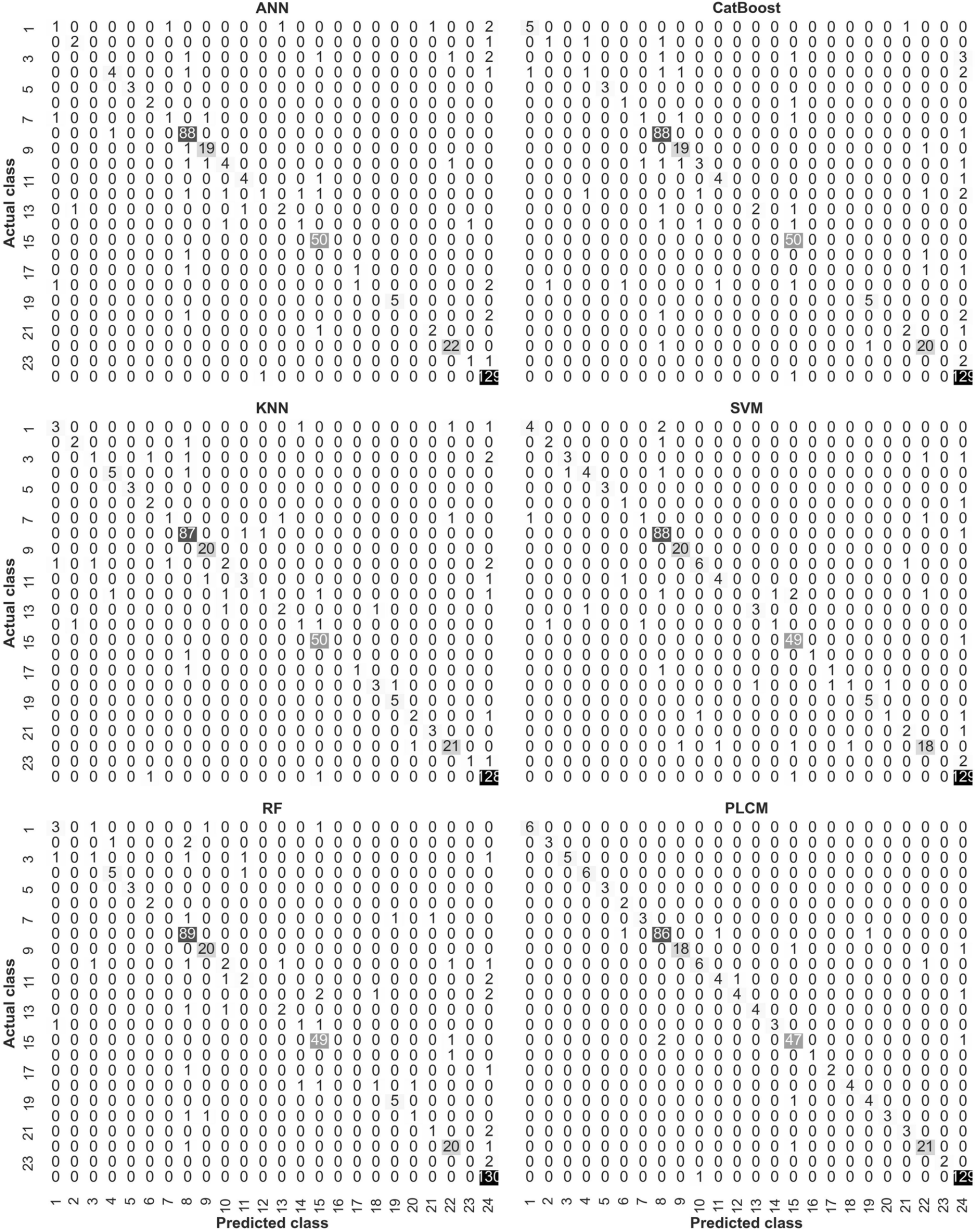
Confusion matrix for all data-driven methods and classes.

The performance of the individual methods and the PLCM method on each class is revealed by the confusion matrix, ROC curve, and precision-recall curve. The confusion matrix is illustrated in Fig. 8, which shows the number of correct and incorrect predictions by each data-driven method for each EOR technique (class) on the test data. The EOR methods are encoded to numeric values. Each row represents a unique class and the sum of values in each row represents the total number of samples belonging to that class. For example, the total number of class 24 (steam injection) in the test subset is 130. As shown in Fig. 8, all methods have approximately the same performance on classes with a high number of samples. For example, all predictive methods have the highest number of true positives on classes 8, 9, 15, 22, and 24, which is the reason for the high accuracy of the methods. However, the main difference between them is the number of correct predictions on rare classes, that is reflected in the precision and recall values. One of the rare classes is class 3 with only 5 samples in the testing subset. For this class, the ANN had zero, the CatBoost had zero, the KNN had one, the SVM had three, and the RF had only one true positive(s). There are also other rare classes that the individual methods had the identical performance as on class 3. This indicates the inability of the individual methods to handle the class-imbalance issue. This issue is resolved by the PLCM method as shown in the bottom-right confusion matrix. For example, this method could predict the correct class of all five samples of class 3. Also, some other examples of rare classes are class 21 and class 20, where the PLCM method had the correct predictions for all samples.

**Figure 9:**
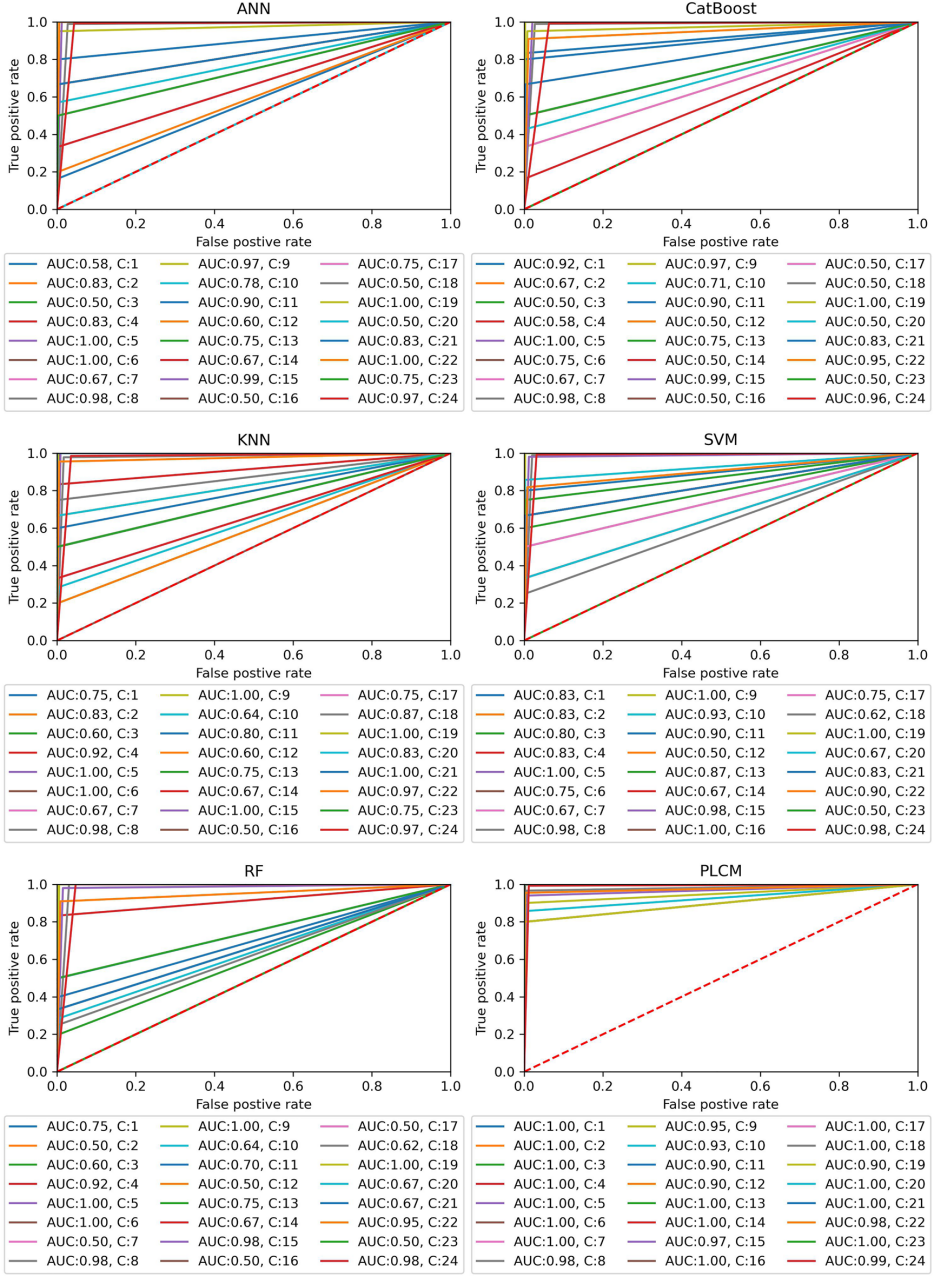
ROC curves for all data-driven methods and classes.

To have a more convenient way of comparing the ability of different predictive methods to handle the class-imbalance issue, the ROC and Precision-Recall curves are used. Figure 9 shows the ROC curves for all methods and classes. Also, the AUC values are reported under each plot. As shown in Fig. 9, the individual methods have resulted in many ROC curves close to the diagonal line, indicating their weak performance on those classes. According to the reported AUCs and class numbers below each plot, the low AUC values are obtained on rare classes. However, this problem is not seen on the ROC curve of the PLCM method. Most of the curves are close to the top-left corner of the plot and AUC values are above 0.95, except for class 10. This shows the great capability of the PLCM method to capture the positive classes while identifying the negative classes correctly.

**Figure 10:**
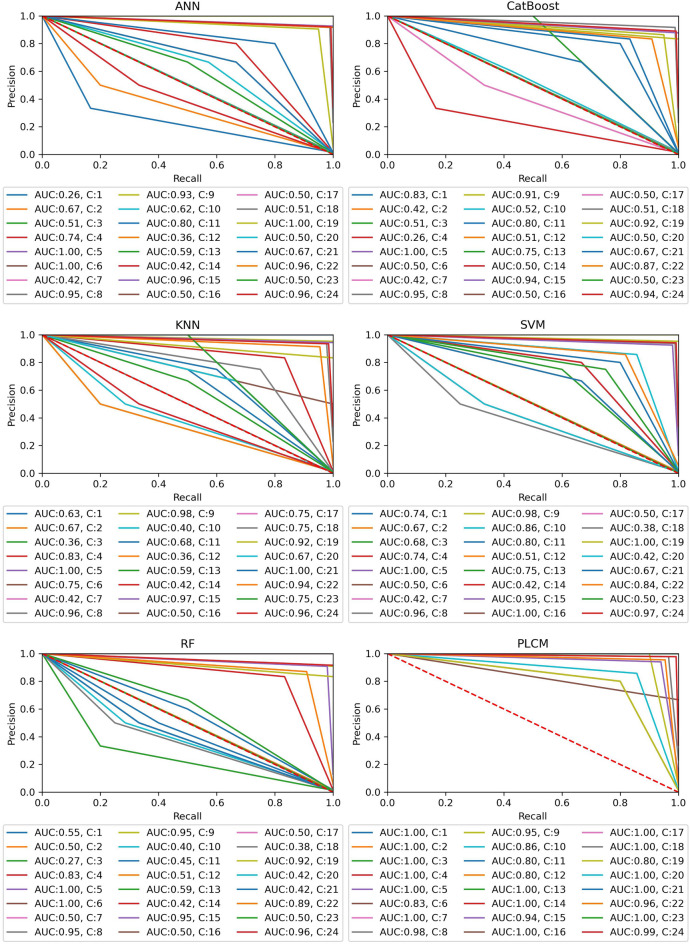
Precision-Recall curves for all data-driven methods and classes.

Figure 10 illustrates the Precision-Recall curve and AUCs for all methods and classes. Same as the ROC curves, the individual methods exhibit many curves far from the optimal point of the plot (top-right corner). The reported AUCs reveal that the pitfall of the individual methods are the rare classes. Nonetheless, the PLCM method has resolved this issue with Precision-Recall curves close to the top-right corner. This method has achieved AUCs of 1 for most of the rare classes and there are only few rare classes that have AUCs below 1. However, all AUCs are above 0.80. Consequently, combining the individual methods by the PLCM method has effectively increased the precision and recall of the model simultaneously.

### Sensitivity analysis

In the end, a sensitivity analysis is conducted to obtain the features importance. To do so, the Pearson’s correlation coefficient^62^ is used. This method determines the importance of each feature, denoted by $r_{j} ,$ known as the relevancy factory. The relevancy factor varies between − 1 and 1. A relevancy factor of 1 indicates a strong direct relationship between a feature and the output, while a relevancy factor of − 1 indicates a strong inverse relationship. The relevancy factor for a feature is defined as

22 $$r_{j} = \frac{{\mathop \sum \nolimits_{i = 1}^{M} \left( {X_{j,i} - \overline{X}_{j} } \right)\left( {y_{i} - \overline{y}} \right)}}{{\sqrt {\mathop \sum \nolimits_{i = 1}^{M} \left( {X_{j,i} - \overline{X}_{j} } \right)^{2} \mathop \sum \nolimits_{i = 1}^{n} \left( {y_{i} - \overline{y}} \right)^{2} } }}, \quad for \;\;(j = 1,\; \ldots ,\;n)$$

where $\overline{X}_{j}$ is the average of feature $j$ over the entire dataset, $y_{i}$ is the output of sample $i$, and $\overline{y}$ is the average of all outputs.

**Figure 11:**
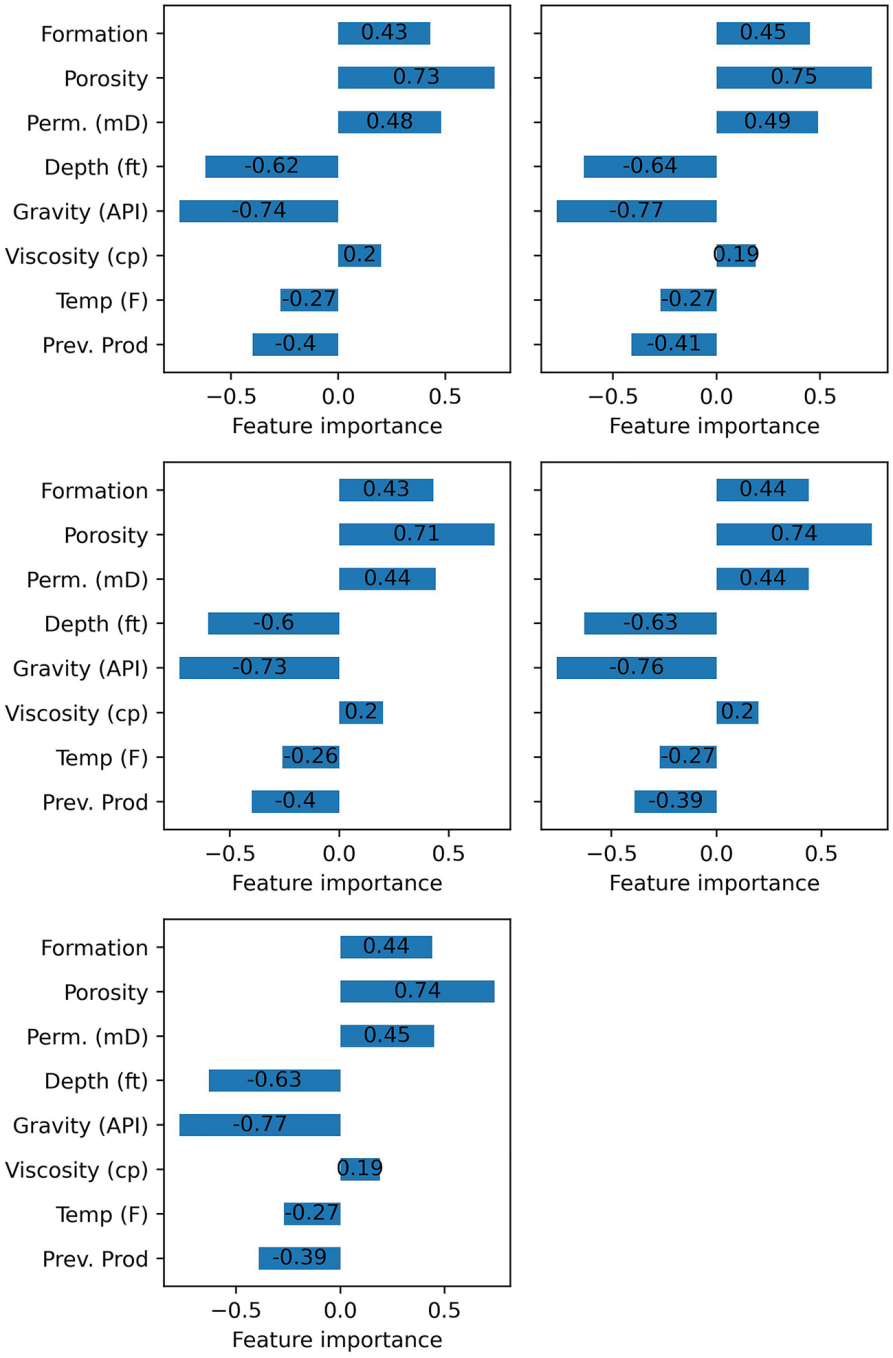
Importance of the features for the individual methods.

Figure 11 demonstrates the feature importance (relevancy factor) of the features for the individual data-driven methods. All methods show the highest sensitivity to oil gravity and porosity with an average relevancy factor of − 0.76 and 0.73, respectively. The depth of the reservoir shows a relatively high impact on the performance of the models with an average importance of − 0.63. Then, permeability, formation type, and the previous production mechanism show a moderate sensitivity with an average relevancy factor of 0.46, 0.44, and − 0.40, respectively. Finally, the temperature and viscosity have the least importance with average importance of − 0.26 and − 0.20, respectively.

The reason for the high impact of the oil gravity on the screening of EOR techniques is that the oil gravity affects the density difference between the injected fluid into the reservoir and the oil in-place. The density difference is one of the most influential factors at designing EOR projects. Porosity is mainly connected to the storage capacity (oil-in-place), capillary pressure, rock and fluid interaction, and fluid flow. Capillary pressure determines the ability of fluids to flow through small pores and into smaller capillaries. Capillary forces can facilitate the oil displacement from small pores, but it can also cause trapping oil in larger pores^63^. The oil-in-place is one of the main factors for the success of any EOR project. Higher porosity means higher oil-in-place volume and higher revenue from the project. In addition, higher porosity generally provides a larger pathway to the injected fluid, leading to a better sweep efficiency. Depth of the reservoir is also a crucial factor that affects the success of an EOR technique. For example, depth is a limiting factor for steam injection since the steam can cool down before reaching the desired depth. Temperature and oil viscosity show the least importance among the input variables. This is because different EOR techniques can be applied to different reservoirs with similar temperature and oil viscosity. For example, water-flooding, steam injection, in-situ combustion, hot water injection, immiscible gas injection (CO_2_ or hydrocarbon gas) are applicable within a wide range of viscosities and temperatures.

### Computational cost and complexity

In addition to the accuracy of the models, the computational cost and complexity of the models should be taken into account. Computational complexity indicates how the computational cost of an algorithm scales with the size of the inputs and samples. It is a measure of the required resources, such as time, memory, or energy by algorithms. This subsection summarizes the computational cost and complexity of the utilized methods. The computational complexity of the models is calculated as follows:

- ANN^64^:
  Training complexity = $O\;(M \times N \times h \times T)$, where $M$ is the number of training samples, $N$ is the number of features, $h$ is the number of hidden neurons, and $T$ is the number of epochs.
  Prediction complexity = $$O\;(M \times N \times h)$$.
- RF^65^:
  Training complexity = $O(M \times N \times \log (N) \times T)$, where $T$ is the number of trees.
  Prediction complexity = $$O(N \times \log (T))$$.
- CatBoost^38^:
  Training complexity = $O\;(M \times N \times \log (N) \times T)$, where $T$ is the number of trees.
  Prediction complexity = $$O\;(N \times \log (T))$$.
- SVM^66^:
  Training complexity = $$O(M^{2} \times N)$$.
  Prediction complexity = $O(N \times n_{s} )$, where $n_{s}$ is the number of support vectors.
- KNN^39^:
  Training complexity = brute force: $O\left( 1 \right)$, as it only stores the training examples, KD Tree: $O\;(M \times N \times \log M)$, Ball Tree: $O\;(M \times N \times \log M)$.
  Prediction complexity = brute force: $O\;(M \times N \times K)$, KD Tree: $O\;(K \times \log \;M)$, Ball Tree: $O\;(K \times \log \;M)$.
- PLCM:
  Training complexity = $O\;(M \times P \times It)$, where $P$ and $It$ is the number of particles and iterations in the PSO.
  Prediction complexity $$O\;(M)$$.

Since the PLCM needs training the individual methods, its overall complexity is the sum of the complexity of the individual methods and the complexity of training and testing of the PLCM. Similarly, its overall prediction complexity is the sum of the prediction complexity of the individual methods and its own prediction complexity.

Table 7 reports the computational cost of training and testing of the employed models. The computational cost is represented by the CPU time needed to train and test the models. Training also includes the tuning of the hyperparameters. As shown in Table 7, the ANN has the highest computational costs due to its enormous number of trainable weights/parameters. However, all methods have a negligible testing/inference time. Among the methods, the CatBoost and PLCM have shown the fastest inference with a CPU time of 0.2 s. Although the PLCM method requires the training of individual methods, its own computational cost is negligible. On the other hand, generally, we train some data-driven methods to solve the classification tasks. Thereby, the only thing we have to do is optimizing the weights of the PLCM method to combine the outputs of those models. Consequently, we can exclude the computational cost of the individual methods from the computational cost of the PLCM. All runs were conducted on an Intel Core i7-7500U CPU with 12 GB RAM.

**Table 7:** Training and testing computational cost of the utilized data-driven methods.

	KNN	RF	CatBoost	SVM	ANN	PLCM
Train (s)	7	543	285	120	5470	6428 + 3
Test (s)	0.27	0.40	0.20	0.81	1.42	3.1 + 0.2

This study showed the strong ability of the PLCM method to effectively combine the outcome of individual EOR screening methods to tackle the class-imbalance issue, which is common in EOR screening problems. This is important because identifying rare EOR methods is difficult, and suggesting wrong EOR techniques for a candidate reservoir may result in severe damages to the reservoir or financial losses. Therefore, this method is recommended as a quick tool to evaluate different EOR techniques for a specific field. Since this study has used a larger dataset sampled from a greater number of countries than any previous work in the field of EOR screening, the developed model is more generalizable to unseen cases around the world.

One of the limitations of this work is that it uses the average reservoir characteristics (permeability, porosity, etc.); however, these properties are heterogeneous and significantly impact the fluid flow in porous media. Also, the current study did not take into account economic factors, which is an important parameter in assessing the success of an EOR project. One of the challenges in developing the models is tuning the hyperparameters. Because of the large number of hyperparameters for each model, high computational resources should be used to have a more comprehensive investigation of these parameters.

It is suggested to consider the heterogeneity of the reservoir properties to model EOR screening in future works. Including more samples and more reservoir features for model development would be helpful.

## Conclusions

The power-law committee machine (PLCM) coupled with the particle swarm optimization (PSO) was used to merge the predictions of five machine learning models, including an ANN, SVM, RF, KNN, and CatBoost, to resolve the class-imbalance problem in classification of EOR techniques. The machine learning models and the proposed method were trained, validated, and tested using 2563 successful real-field EOR applications around the world. The individual machine learning models could achieve an average F1-Score of 0.868, where the KNN and SVM had the highest F1-Scores with the values of 0.894 and 0.892, respectively. In addition, the individual methods achieved an average accuracy, precision, and recall of 0.891, 0.850, 0.891, respectively, indicating their inability to handle the class-imbalance issue. The low value of the precision revealed that the utilized machine learning methods lacked rigor to correctly predict positive classes as positives. The novelty of this paper is to utilize the PLCM method to combine the outputs of the individual machine learning methods to tackle the class-imbalance issue by taking benefit from the strength point of each individual method. The results show that the proposed method could effectively combine the predictions by different models to overcome the class-imbalance challenge. The F1-Score on the test subset increased to 0.963, and the accuracy, precision, and recall improved to 0.963, 0.964, and 0.963, respectively, indicating the ability of the proposed model to correctly identify the positive classes (high precision) while having a high rate of true positives (recall), which was shown in the ROC and precision-recall curves. A sensitivity analysis was conducted in this study, which found the oil gravity and the formation porosity as the most influential parameters on screening of the EOR techniques. This is because the gravity of the oil greatly affects the density difference between the injected and formation fluids, influencing the macroscopic sweep efficiency. Also, the formation porosity is related to the oil-in-place, fluid flow, capillary pressure, and rock and fluid interactions, where these parameters significantly determine the success of an EOR project. On the other hand, temperature and viscosity had the least importance since a variety of EOR techniques can be applied to different reservoirs with similar temperature and oil viscosity. Finally, this paper showed that the PLCM is a fast and accurate model to predict the suitable EOR technique for oilfields. Therefore, reservoir engineers can have a quick evaluation of the EOR technique for a specific field without spending too much time and financial resources.

### Supplementary Information

Supplementary Information.

## Data Availability

Data is provided within the manuscript or supplementary information files.

## Abbreviations

AEORS: Advanced enhanced oil recovery screening
ANN: Artificial neural network
AUC: Area under the curve
CEORS: Conventional enhanced oil recovery screening
CPU: Central processing unit
EOR: Enhanced oil recovery
FN: False negative
FP: False positive
FPR: False positive rate
KNN: K-nearest neighbors
LPG: Liquefied petroleum gas
MLP: Multi-layer perceptron
NB: Naïve Bayes
PLCM: Power-law committee machine
PSO: Particle swarm optimization
RAM: Random access memory
RF: Random forest
ROC: Receiver operating characteristic
SAGD: Steam assisted gravity drainage
SGD: Stochastic gradient descent
SVM: Support vector machine
TN: True negative
TP: True positive
TPR: True positive rate

## References

[1] Zhang C (2022). Mechanism for the formation of natural fractures and their effects on shale oil accumulation in Junggar Basin, NW China. Int. J. Coal Geol..

[2] Cui K (2019). Stimulation of indigenous microbes by optimizing the water cut in low permeability reservoirs for green and enhanced oil recovery. Sci. Rep..

[3] Vo Thanh H, Sugai Y, Sasaki K (2020). Application of artificial neural network for predicting the performance of CO_2_ enhanced oil recovery and storage in residual oil zones. Sci. Rep..

[4] Wang X (2023). Mechanism of enhanced oil recovery by fuzzy-ball fluid as a novel oil-displacement agent. Energy Rep..

[5] Mahdaviara M, Sharifi M, Ahmadi M (2022). Toward evaluation and screening of the enhanced oil recovery scenarios for low permeability reservoirs using statistical and machine learning techniques. Fuel.

[6] Cheraghi Y, Kord S, Mashayekhizadeh V (2021). Application of machine learning techniques for selecting the most suitable enhanced oil recovery method; challenges and opportunities. J. Pet. Sci. Eng..

[7] Xiao D (2023). Model for economic evaluation of closed-loop geothermal systems based on net present value. Appl. Therm. Eng..

[8] Syed FI, Muther T, Dahaghi AK, Neghabhan S (2022). CO_2_ EOR performance evaluation in an unconventional reservoir through mechanistic constrained proxy modeling. Fuel.

[9] Shen B, Yang S, Chen H, Li S, Gao X (2022). A novel CO_2_-EOR potential evaluation method based on Bo-Lightgbm algorithms using hybrid feature mining. SSRN Electron. J..

[10] Chavan HK, Sinharay RK, Kumar V, Patel D (2023). An approach of using machine learning classification for screening of enhanced oil recovery techniques. Pet. Sci. Technol..

[11] Bera A, Vij RK, Shah S (2021). Impact of newly implemented enhanced oil and gas recovery screening policy on current oil production and future energy supply in India. J. Pet. Sci. Eng..

[12] Yang L, Wang H, Xu H, Guo D, Li M (2023). Experimental study on characteristics of water imbibition and ion diffusion in shale reservoirs. Geoenergy Sci. Eng..

[13] Zhang R (2017). Major factors controlling fracture development in the Middle Permian Lucaogou Formation tight oil reservoir, Junggar Basin, NW China. J. Asian Earth Sci..

[14] Kumar Pandey R, Gandomkar A, Vaferi B, Kumar A, Torabi F (2023). Supervised deep learning-based paradigm to screen the enhanced oil recovery scenarios. Sci. Rep..

[15] Syafitri N, Arta Y (2022). Fuzzy-based screening system for determination of enhanced oil recovery (EOR) method in reservoir. IT J. Res. Dev..

[16] Khazali N, Sharifi M, Ahmadi MA (2019). Application of fuzzy decision tree in EOR screening assessment. J. Pet. Sci. Eng..

[17] Giro, R., Lima Filho, S. P., Neumann Barros Ferreira, R., Engel, M. & Steiner, M. B. Artificial Intelligence-Based Screening of Enhanced Oil Recovery Materials for Reservoir-Specific Applications. *Offshore Technology Conference Brasil* D031S033R005 at 10.4043/29754-MS (2019).

[18] Su S (2023). Investigation and optimization of EOR screening by implementing machine learning algorithms. Appl. Sci..

[19] Tabatabaei SM, Attari N, Panahi SA, Asadian-Pakfar M, Sedaee B (2023). EOR screening using optimized artificial neural network by sparrow search algorithm. Geoenergy Sci. Eng..

[20] Khojastehmehr M, Madani M, Daryasafar A (2019). Screening of enhanced oil recovery techniques for Iranian oil reservoirs using TOPSIS algorithm. Energy Rep..

[21] Hien DH, Long H, Ngoc PQ (2021). Screening selection of enhanced oil recovery methods based on analytics of worldwide oilfield data with reference to offshore oil fields in Vietnam. Petrovietnam J..

[22] Sheng JJ (2013). Enhanced Oil Recovery Field Case Studies.

[23] Moritis. 1998 Worldwide EOR Survey. *Oil Gas J.* (1998).

[24] Ma, R., Kong, D., Wang, F., Xin, X. & Li, Y. Oil Production Plant of Daqing Oilfield Company, Petro China; Zhengbo Wang, Research Institute of Petroleum Exploration and Development, Petro China; Huifeng Liu, Tarim Oil Company, Petro China; the SPE Improved Oil Recovery Conference. *Jiayu Dong* 14–18 (2018).

[25] Koottungal. 2012 Worldwide EOR Survey. *Oil Gas J.* (2012).

[26] Koottungal, L. 2014 Worldwide EOR Survey. *Oil Gas J.* (2014).

[27] Worldwide. 2004 Worldwide EOR Survey. *Oil Gas Facil.* (2004).

[28] Yousefzadeh R, Bemani A, Kazemi A, Ahmadi M (2022). An insight into the prediction of scale precipitation in harsh conditions using different machine learning algorithms. SPE Prod. Oper..

[29] Vakili-Nezhaad GR, Al Shaaili A, Yousefzadeh R, Kazemi A, Al Ajmi A (2024). CO_2_-brine interfacial tension correlation based on the classical orthogonal polynomials: Monovalent salts with common anion. Chem. Pap..

[30] Luo J, Wang Y, Li G (2023). The innovation effect of administrative hierarchy on intercity connection: The machine learning of twin cities. J. Innov. Knowl..

[31] Ng CSW, Djema H, Nait Amar M, JahanbaniGhahfarokhi A (2022). Modeling interfacial tension of the hydrogen-brine system using robust machine learning techniques: Implication for underground hydrogen storage. Int. J. Hydrog. Energy.

[32] Amar MN, Ouaer H, AbdelfetahGhriga M (2022). Robust smart schemes for modeling carbon dioxide uptake in metal–organic frameworks. Fuel.

[33] Gholami M, Ranjbargol M, Yousefzadeh R, Ghorbani Z (2023). Integrating three smart predictive models using a power-law committee machine for the prediction of compressive strength in masonry made of clay bricks and cement mortar. Structures.

[34] Juna A (2022). Water quality prediction using KNN imputer and multilayer perceptron. Water.

[35] Goodfellow I, Bengio Y, Courville A (2016). Deep Learning.

[36] Shahani NM, Zheng X, Guo X, Wei X (2022). Machine learning-based intelligent prediction of elastic modulus of rocks at thar coalfield. Sustainability.

[37] Vaferi B, Dehbashi M, Hosin A, Yousefzadeh R (2024). Exploring the performance of machine learning models to predict carbon monoxide solubility in underground pure/saline water. Mar. Pet. Geol..

[38] Prokhorenkova, L., Gusev, G., Vorobev, A., Dorogush, A. V. & Gulin, A. CatBoost: Unbiased boosting with categorical features. In *Proceedings of the 32nd International Conference on Neural Information Processing Systems* 6639–6649 (2018).

[39] Fix E, Hodges JL (1989). Discriminatory analysis. Nonparametric discrimination: Consistency properties. Int. Stat. Rev. Int. Stat..

[40] Piryonesi SM, El-Diraby TE (2020). Role of data analytics in infrastructure asset management: Overcoming data size and quality problems. J. Transp. Eng..

[41] Brajard J, Carrassi A, Bocquet M, Bertino L (2020). Combining data assimilation and machine learning to emulate a dynamical model from sparse and noisy observations: A case study with the Lorenz 96 model. J. Comput. Sci..

[42] Cortes C, Vapnik V (1995). Support-vector networks. Mach. Learn..

[43] Shao M, Wang X, Bu Z, Chen X, Wang Y (2020). Prediction of energy consumption in hotel buildings via support vector machines. Sustain. Cities Soc..

[44] Ahmad MS, Adnan SM, Zaidi S, Bhargava P (2020). A novel support vector regression (SVR) model for the prediction of splice strength of the unconfined beam specimens. Constr. Build. Mater..

[45] Zhang, H. *et al.* Combining machine learning and classic drilling theories to improve rate of penetration prediction. In *Proc. SPE/IADC Middle East Drill. Technol. Conf. Exhib.*10.2118/202202-ms (2021).

[46] Amar MN, Zeraibi N, JahanbaniGhahfarokhi A (2020). Applying hybrid support vector regression and genetic algorithm to water alternating CO_2_ gas EOR. Greenh. Gases Sci. Technol..

[47] Amar MN, Zeraibi N (2019). A combined support vector regression with firefly algorithm for prediction of bottom hole pressure. SN Appl. Sci..

[48] Takhanov R (2023). On the speed of uniform convergence in Mercer’s theorem. J. Math. Anal. Appl..

[49] Ahamed, H., Alam, I. & Islam, M. SVM Based Real Time Hand-Written Digit Recognition System (2019).

[50] Ahlawat S, Choudhary A (2020). Hybrid CNN-SVM classifier for handwritten digit recognition. Procedia Comput. Sci..

[51] Yan T, Xu R, Sun S-H, Hou Z-K, Feng J-Y (2023). A real-time intelligent lithology identification method based on a dynamic felling strategy weighted random forest algorithm. Pet. Sci..

[52] Anmala J, Turuganti V (2021). Comparison of the performance of decision tree (DT) algorithms and extreme learning machine (ELM) model in the prediction of water quality of the Upper Green River watershed. Water Environ. Res..

[53] Fang X (2021). Random forest-based understanding and predicting of the impacts of anthropogenic nutrient inputs on the water quality of a tropical lagoon. Environ. Res. Lett..

[54] Kennedy, J. & Eberhart, R. Particle swarm optimization. In *Proceedings of ICNN’95—International Conference on Neural Networks* 1942–1948. 10.1109/ICNN.1995.488968 (1995).

[55] Yousefzadeh, R., Kazemi, A., Ahmadi, M. & Gholinezhad, J. *Introduction to geological uncertainty management in reservoir characterization and optimization : robust optimization and history matching.* (Springer Cham, 2023).

[56] Poli R, Kennedy J, Blackwell T (2007). Particle swarm optimization. Swarm Intell..

[57] Yousefzadeh R, Ahmadi M, Kazemi A (2022). Toward investigating the application of reservoir opportunity index in facilitating well placement optimization under geological uncertainty. J. Pet. Sci. Eng..

[58] Yousefzadeh R, Sharifi M, Rafiei Y (2021). An efficient method for injection well location optimization using fast marching method. J. Pet. Sci. Eng..

[59] Yousefzadeh R, Ahmadi M (2023). Fast marching method assisted permeability upscaling using a hybrid deep learning method coupled with particle swarm optimization. Geoenergy Sci. Eng..

[60] Ding S, Lu R, Xi Y, Liu G, Ma J (2020). Efficient well placement optimization coupling hybrid objective function with particle swarm optimization algorithm. Appl. Soft Comput..

[61] Sharifipour M, Nakhaee A, Yousefzadeh R, Gohari M (2021). Well placement optimization using shuffled frog leaping algorithm. Comput. Geosci..

[62] Boslaugh S, Watters PA (2008). Statistics in a Nutshell: A Desktop Quick Reference.

[63] Xu Z (2022). Characteristics of Source Rocks and Genetic Origins of Natural Gas in Deep Formations, Gudian Depression, Songliao Basin, NE China. ACS Earth Sp. Chem..

[64] Nielsen M (2015). Neural Networks and Deep Learning.

[65] James G, Witten D, Hastie T, Tibshirani R, James G, Witten D, Hastie T, Tibshirani R (2013). Tree-Based Methods BT—An Introduction to Statistical Learning: With Applications in R.

[66] James G, Witten D, Hastie T, Tibshirani R, James G, Witten D, Hastie T, Tibshirani R (2013). Support Vector Machines BT—An Introduction to Statistical Learning: With Applications in R.

